# Contribution to the Chemotherapy of Human Trypanosomiasis: Design, Synthesis, and Biological Evaluation of Dimeric 2‑Nitroimidazoles against *Trypanosoma cruzi* Amastigotes and Bloodstream *Trypanosoma brucei*

**DOI:** 10.1021/acsomega.5c09284

**Published:** 2025-12-26

**Authors:** Afonso Santine M. M. Velez, Otávio Augusto Chaves, Carlos Serpa, Fatma M. Salem, Bibo Li, Bin Su, Célio Geraldo Freire-de-Lima, Debora Decote-Ricardo, Marco Edilson Freire de Lima

**Affiliations:** † Programa de Pós-graduação Em Química, Instituto de Química, Universidade Federal Rural Do Rio de Janeiro, Seropédica, Rio de Janeiro 23.897-000, Brazil; ‡ Department of Chemistry, Coimbra Chemistry Center - Institute of Molecular Science (CQC-IMS), 37829 University of Coimbra, Rua Larga, Coimbra 3004-535, Portugal; § Laboratory of Immunopharmacology, Centro de Pesquisa Inovação E Vigilância Em COVID-19 E Emergências Sanitárias (CPIV), Oswaldo Cruz Institute (IOC), Rio de Janeiro, Rio de Janeiro 21040-361, Brazil; ∥ Department of Chemistry, College of Sciences and Health Professions, 2564 Cleveland State University, 2121 Euclid Avenue, Cleveland, Ohio 44115, United States; ⊥ Department of Biology, Geo. & Env. Sciences, College of Sciences and Health Professions, 170252 Cleveland State University, 2121 Euclid Avenue, Cleveland, Ohio 44115, United States; # Instituto de Biofísica Carlos Chagas Filho, Universidade Federal Do Rio de Janeiro, Rio de Janeiro, Rio de Janeiro 21.941-902, Brazil; ∇ Departamento de Microbiologia E Imunologia Veterinária, Instituto de Veterinária - Universidade Federal Rural Do Rio de Janeiro, Seropédica, Rio de Janeiro 23897-035, Brazil

## Abstract

Effective and safe treatments for neglected tropical diseases caused by parasites, such as Chagas disease and sleeping sickness, remain lacking, posing a significant challenge for researchers worldwide. The rational design of dimeric compounds inspired solely by the pharmacophoric core of benznidazole (2-nitroimidazole) has proven to be a promising strategy for antiparasitic development. Thus, in the present work, it was increased the linker between the active units (2-nitroimidazole) to improve the interaction with TcNTR, facilitating the bioactivation of the longest dimers. Biological assays confirmed this, demonstrating that all compounds were active against replicative intracellular amastigotes of *Trypanosoma cruzi* (Tulahuen C2C4-*LacZ*). Notably, longer-chain dimers exhibited remarkable potency (IC_50_ < 1.0 μM). These compounds also showed significant activity against *T. b. brucei* and demonstrated very low cytotoxicity in mammalian cells, highlighting their selectivity, especially among the longer-chain dimers. These findings support the development of dimeric 2-nitroimidazole derivatives as selective agents against trypanosomes.

## Graphical abstract

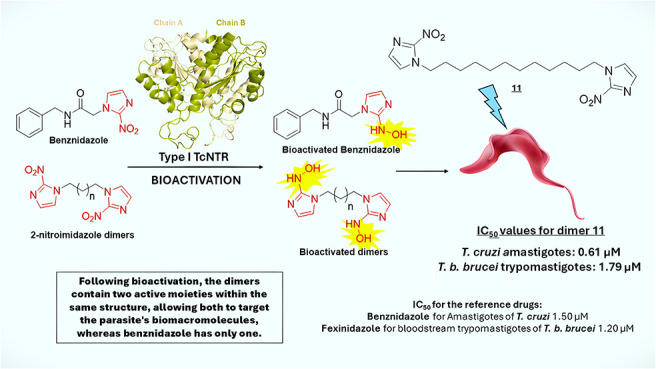

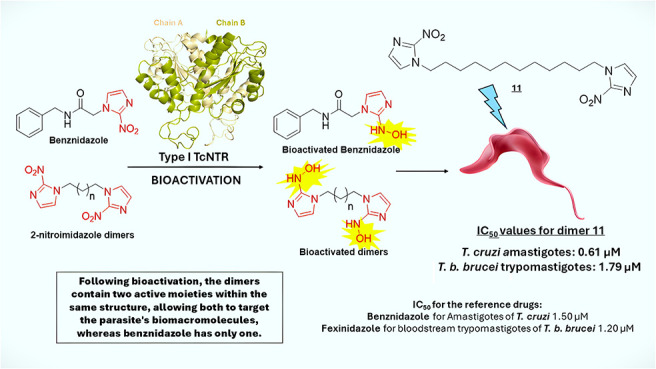

## 1 Introduction

The primary human trypanosomiases of medical and public health concerns within endemic regions are Chagas disease (American trypanosomiasis, CD) and sleeping sickness (Human African trypanosomiasis, HAT). Both represent parasitic infections caused by hemoflagellate protozoa belonging to the Trypanosomatidae family and the *Trypanosoma* genus.^1,2^ These infections are predominantly transmitted via insect vectors, i.e., HAT through the tsetse fly (*Glossina* spp.), while CD via triatomine bugs, commonly known as kissing bugs, including *Triatoma infestans*, *Rhodnius prolixus*, and *Panstrongylus megistus*.

Sleeping sickness results from infection by two subspecies of *Trypanosoma brucei* (*T. brucei*): *T. b. gambiense* (g-HAT, accounting for approximately 98% of reported cases) and *T. b. rhodesiense* (r-HAT). The disease is a neurological disorder of the central nervous system that primarily affects Africa. In the advanced stage, infection caused by *T. brucei* leads to meningoencephalitis, resulting in severe neuropsychiatric changes. Furthermore, cardiac impairment, such as perimyocarditis, is also documented.^3^ Absent adequate intervention, these clinical conditions may become critical, potentially leading to mortality. Conversely, CD is caused by *Trypanosoma cruzi* (*T. cruzi*) being widespread across Latin America, impacting the cardiac and digestive systems as patients advance into the chronic phase, characterized by hypertrophy of vital organs and compromised functionality.^4,5^ Many patients with chronic CD ultimately succumb to this condition.

The World Health Organization (WHO) classifies these parasitic diseases as neglected tropical diseases (NTDs), as they are socially determined illnesses that affect populations living in impoverished areas characterized by high economic and social vulnerability, where people have limited access to basic sanitation or formal public health systems. According to the WHO, over 7 million people are infected with *T. cruzi*. Additionally, nearly seventy-five million individuals reside in regions at risk of infection. Furthermore, the population living in endemic areas affected by the disease and have not yet received an adequate diagnosis constitutes a significant number 70%.^6–8^ Another factor to consider is that major pharmaceutical corporations do not allocate sufficient resources to the search for and development of new treatments for these parasitic diseases. Essentially, there is a humanitarian need, but no market.

The *T. cruzi* exhibits three morphological forms during its life cycle, i.e., the epimastigote, a replicative form found in the digestive tract of the triatomine insect; the trypomastigote, an infective and nonreplicating form present in the bloodstream of the mammalian host and in the wastes of the triatomine insect; and the amastigote, a replicative form observed inside mammalian cells. The amastigote form is considered the most clinically significant due to its central role in maintaining the infection and promoting parasite proliferation in human hosts.^7,10^ Furthermore, in the case of *T. brucei*, the parasite’s life stages are distributed between invertebrate hosts (*Glossinia* spp.) and vertebrates (humans, primates, and ungulate mammals such as zebras, horses, cattle, and goats). During a blood meal, the vector injects metacyclic trypomastigote forms, which are present in its salivary glands, into the vertebrate host. Once within the host’s circulatory system, the parasite differentiates into bloodstream trypomastigotes and migrates to other compartments and bodily fluids, such as lymph and cerebrospinal fluid. In contrast to *T. cruzi*, all life stages of *T. brucei* are extracellular, with the bloodstream trypomastigote form being the most clinically significant.^9^

Current chemotherapy for CD relies on two nitroheterocyclic drugs: nifurtimox (Figure 1) and benznidazole (compound 1, Figure 1). These drugs demonstrate cure rates of 50% to 70% during the acute phase, compared to rates of less than 20% in the chronic phase.^4,10,11^ These medicines require lengthy treatment durations of 30–60 days, which can lead to low adherence and potential discontinuation of therapy. Serious adverse effects are a key concern.^12–14^ Additionally, HAT therapy involves highly cytotoxic drugs that can lead to severe adverse effects, ultimately deterring their use. Most of these drugs are administered intramuscularly and intravenously, which can be painful and also expensive, and have limited availability in endemic areas due to their low-temperature storage requirements.^9^ These treatment methods often fail to achieve an adequate cure rate due to the rapid progression of HAT’s clinical presentation or the serious adverse effects. Currently used medications include fexinidazole (compound **2**), pentamidine, melarsoprol, eflornithine, and suramin (Figure 1), used alone or in combination with nifurtimox. Treatment is tailored to the stage of the disease and the *T. brucei* species, emphasizing the critical importance of accurate diagnosis. These factors complicate treatment and increase its cost for this potentially fatal disease. Thus, there is an apparent demand for more effective drugs that offer greater selectivity to ensure enhanced safety for infected patients.^9,15,16^

**Figure 1:**
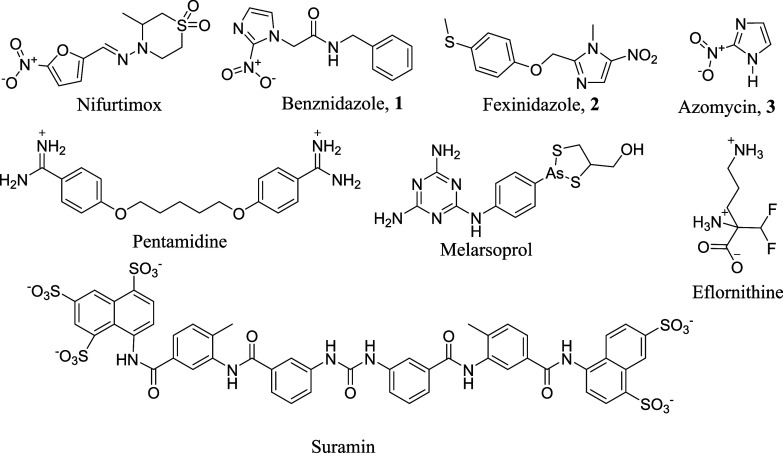
Chemical structures of commercial antiparasitic drugs. Azomycin is a commercial natural antibiotic. For better interpretation, the counterions were omitted.

Although nitroheterocyclic drugs used to treat parasitic diseases have notable limitations—especially related to cytotoxicity and tolerability—they remain essential parts of the current antiparasitic toolkit. Despite its limited effectiveness in the chronic phase of CD, benznidazole remains the preferred treatment, showing reasonable efficacy in children and in the acute phase of infection. However, its significant systemic toxicity emphasizes the need to optimize dosage regimens, explore combination therapies, or develop new uses based on the biologically active motifs present in these molecules.^16,17^ On the other hand, fexinidazole has marked a new era by offering a 10-day oral regimen for human African trypanosomiasis—both gambiense and rhodesiense—eliminating the need for lumbar punctures in many cases and reducing the risk of severe adverse effects typically associated with melarsoprol or injectable treatments.^16,18,19^ Clinical trials published in The Lancet Global Health have demonstrated high effectiveness in patients with mild to moderate disease, with only mild to moderate side effects such as nausea, headache, insomnia, or tremors, and no unexpected safety issues.^16,20^ Thus, despite their limitations, especially the side effects of benznidazole, nitroheterocycles are still the only trypanocidal drugs with proven efficacy and accessible options. Finally, it is essential to highlight that the introduction of fexinidazole represents a vital step forward: it is less toxic, more practical, and has the potential to make a significant impact, particularly in remote areas and among vulnerable children.

Since the nitroimidazole core is the main pharmacophore present in benznidazole, serving as a key structural feature for its antiparasitic activity that is activated by the *T. cruzi* nitroreductase (TcNTR) enzyme to cause irreversible damage to biomacromolecules, different analogues conserving the nitro group have been proposed to improve its antiparasitic profile.^12,17,18,23^ In this context, the dimerization of active fragments has been extensively studied as a molecular design strategy to enhance drug-target interactions and improve pharmacological properties.^17^ The dimerization strategy of a pharmacophoric group is a synthetic approach widely explored in the medicinal chemistry field; however, biologically active dimeric structures can also be found in nature.^22,23^ This approach yields interesting results in the design of dimers with antiparasitic potential, as demonstrated by Barbaras and colleagues (2008), who reported the promising activity of synthetic nostocarboline dimers against *T. brucei* and *Plasmodium falciparum*.^18^ Additionally, in the work of Sijm and colleagues, it was explained that phenylpyrazolone dimers are a new class of anti-*T. cruzi* agents.^19^ It is also possible to find many works that utilize this approach in chemotherapy for certain types of cancer, presenting promising results.^20–22^

The pharmacophore dimerization strategy can impact the potency of the dimeric molecule in different ways, depending on the type of interaction and the receptor’s structure.^17^ The fact that each planned molecule has two nitroimidazole units in its structure, in theory, doubles the concentration of pharmacophores near the biochemical target, increasing the likelihood of the interactions necessary for the biological effects occurring. Considering the presented antiparasitic development concern and the intention to meet the humanitarian demand for new antichagasic drugs, a series of eight homologous dimeric molecules (Figure 2) was *in silico* designed targeting TcNTR, based on *N*-alkylated derivatives of 2-nitro-1*H*-imidazole, a natural antibiotic known as azomycin (Figure 1, compound 3), to be *in vitro* validated subsequently. The chemical structure of compound **3** is the pharmacophore of benznidazole, which exhibits antichagasic activity against *T. cruzi* amastigotes comparable to that of the standard drug.^23^ Before the synthetic dimerization approach, *in silico* predictions were done to explore the effect of spacer chains between two units of the pharmacophore 2-nitroimidazole via physicochemical characteristics of the dimers and their capacity to interact with TcNTR in the presence of the cofactor flavin mononucleotide (FMN). To validate the *in silico* predictions, the designed dimeric compounds **4–11** (Figure 2) were synthesized to evaluate their antiparasitic profile against *T. cruzi* amastigotes—the intracellular replicative form of *T. cruzi* (Tulahuen strain C2C4-*LacZ*). To also assess the capacity of the designed compounds to act against other parasitic strains, *in vitro* assays against bloodstream trypomastigotes of *T. b. brucei* were also conducted.^28^ Finally, the cytotoxicity of the proposed dimeric compounds was also assayed to verify their safety.

**Figure 2:**
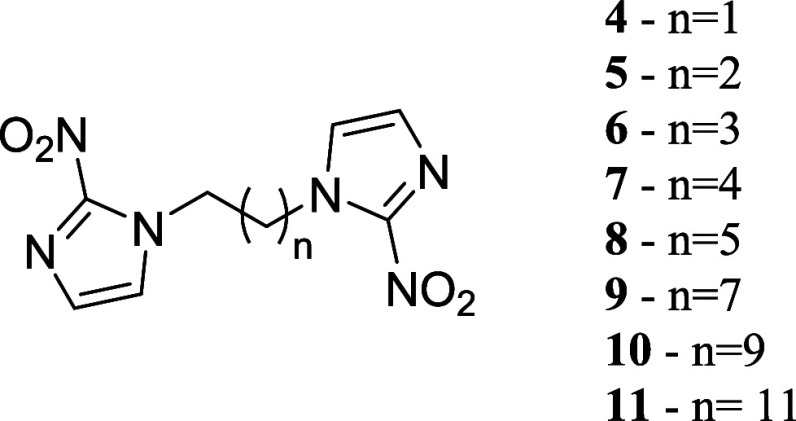
Structures of designed homologous dimeric derivatives of 2-nitroimidazoles (**4–11**).

## 2 Results and Discussion

### 2.1 *In Silico* Drug Design and Antiparasitic Predictions

The dimerization of the pharmacophoric group of the primary drug used in CD treatment, benznidazole (2-nitroimidazole, compound 3 in Figure 1), was planned to explore new molecular design methods for CD therapy. Hall and Wilkinson (2012) explain how benznidazole works inside the parasite cell, where it undergoes intracellular reductions.^23^ Its pharmacophoric group undergoes bioactivation by TcNTR, serving as a prodrug and producing toxic intermediates. This enzyme is found in trypanosomatids and may serve as a drug activator. This process allows the drug to act as a prodrug, as the enzyme catalyzes intracellular nitroreduction reactions that generate various toxic species, including radical intermediates that cause DNA damage in the parasite and lead to apoptosis.^19,34–38^ Variations in the length and conformational flexibility of the spacer chain might be crucial, as they directly influence the activity of these pharmacophores in biological environments. Previously, Tamiz and colleagues (2000) discussed and exemplified this influence on dimeric serotonin inhibitors, highlighting the importance of dimerization in drug design.^24^ Thus, using this approach, we varied the carbon chain from two to 12 methylene units (−CH_2_−) to initially evaluate in silico the impact of carbon chains on the capacity of interaction with TcNTR in the presence of the cofactor FMN.

Since there is no experimental tridimensional structure for TcNTR, an enzymatic model was predicted and validated for *T. cruzi* Tulahuen using as a template the X-ray nitroreductase (NTR) structure from *E. coli* B obtained in the Protein Data Bank with access code 1DS7, following the protocol previously reported by Cirqueira and colleagues—the putative binding site of TcNTR was reported as intact in NTR from *E. coli* B.^25,26^ Figure 3A depicts the obtained predicted homodimeric structure of TcNTR with high superposition of the active site with the reported TcNTR structure from AlphaFold (Figure 3B) within a root-mean-square deviation (RMSD) value of 1.667 Å. Additionally, the predicted TcNTR was also validated by superposition with the reported Swiss-Model TcNTR of *T. cruzi* strain CL Brener (Figure 3B), which is genetically very close to the Tulahuen strain,^27^ with an RMSD value of 1.974 Å and high superposition in the α-helix and β-sheet contents.

**Figure 3:**
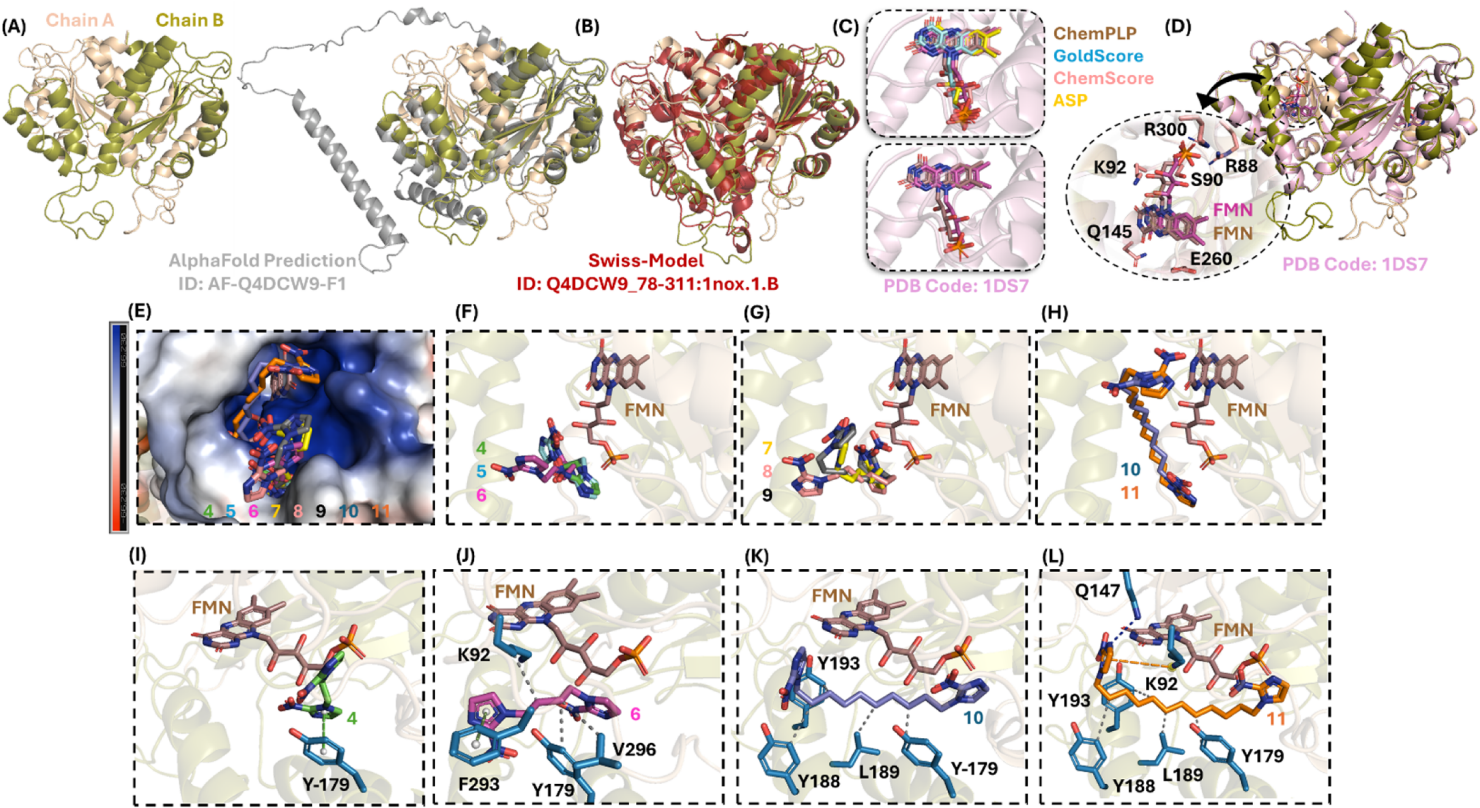
(A) The obtained predicted dimeric model of TcNTR and (B) its superposition with the reported AlphaFold model in gray—predicted Local Distance Difference Test (pLDDT) score for residues across the full-length protein of 61.3% and 19.5%, meaning confidence of very high and high, respectively, and the superposition with the reported predicted Swiss-Model TcNTR from CL Brener in wine. (C) Superposition of the redocking calculations to FMN into the catalytic site of the crystallographic NTR from *E. coli* B. (D) Superposition between the obtained predicted dimeric model of TcNTR and the crystallographic NTR from *E. coli* B. (E) The electrostatic potential map of TcNTR docked with dimeric 2-nitroimidazoles **4–11**. The best docking pose to (F) **4–6**, (G) **7–9**, and (H) **10** and **11** into the catalytic pocket of TcNTR in the presence of the cofactor FMN. The main amino acid residues that interact with (I) **4**, (J) **6**, (K) **10**, and (L) **11**. Blue lines, gray, orange, and green dots represent hydrogen bonds, hydrophobic interactions, π-cation interactions, and π-stacking interactions, respectively. For better interpretation, hydrogen atoms were omitted.

As previously reported by Cirqueira and colleagues, the putative binding site for the enzymatic cofactor FMN into the catalytic pocket of TcNTR is found intact in NTR from *E. coli* B, thus, redocking calculations for FMN (PDB code: 1DS7) were carried out to identify the best scoring functions of GOLD 2025 software (Cambridge Crystallographic Data Center, Cambridge, CB2 1EZ, UK) to predict the binding of FMN to the obtained enzymatic model.^26,28^ As represented in Figure 3C, ChemPLP was identified as the best-scoring function with an RMSD value of 1.053 Å. The docked FMN structure in the TcNTR model shows a high degree of superposition with the experimental one, preserving key interactions with the amino acid residues R88, S90, Q145, E260, and R300 at the dimeric interface (Figure 3D).

Once a TcNTR model was obtained, molecular docking calculations were performed for the designed dimeric products of 2-nitroimidazoles (**4**–**11**) and the precursor reagent **3** to assess their ability to be activated by this enzyme. In the GOLD 2025 software, the docking score value (dimensionless) for each pose accounts for intramolecular tensions within the ligand and intermolecular interactions, and is considered as the negative of the sum of the energy terms involved in the macromolecule–ligand association; therefore, the higher the score, the better the interaction profile.^29^ The docking score values (dimensionless) for **3**, **4**–**11** were 29.8, 50.0, 50.6, 51.4, 55.3, 58.6, 59.7, 67.4, and 62.7, respectively. These results suggest that the designed dimeric products of 2-nitroimidazoles may have greater antiparasitic activity than the precursor reagent **3**. Additionally, there is clear evidence of an improvement in the binding capacity of *in silico* evaluated compounds as the number of methylene units increases.

As depicted in Figure 3E, all dimeric compounds might interact inside the positive electrostatic potential pocket of TcNTR, where the cofactor FMN can be found. It is essential to highlight that the redox reaction within the TcNTR pocket follows a bi-bi ping pong mechanism of kinetics, i.e., the nicotinamide adenine dinucleotide (NADH) is oxidized by the concomitant reduction of FMN to a posterior reduction of nitro compounds by FMNH_2_, thereby regenerating the flavin for further catalytic cycles.^23^ Based on this mechanism, the interaction of **4**–**11** to the enzymatic cofactor is crucial to convert a prodrug into an active drug, and probably due to the increased methylene units in the dimeric compounds improved the kinetic volume (area and volume from 120.7 to 449.2 Å^2^ and from 95.7 to 400.8 Å^3^, respectively, Table 1), flexibility, and the probability of the nitro group to interact with the cofactor as summarized in Figure 3F (**4**, **5**, and **6**), Figure 3G (**7**, **8**, and **9**), and Figure 3H (**10** and **11**), being supported by the increasing values of ovality, from 1.19 to 1.71 (Table 1, the dimeric compounds are more elongated or cigar-shaped to interact with the enzymatic cofactor with the increasing of methylene units).

**Table 1:** Predicted (*In Silico*) Physicochemical Properties and Drug-Likeness for the Dimers Using the Online Web Server SwissADME^32,33^ and Spartan’14 Software

Compound	*n*(CH_2_)	Log *P*_o/w_ ^a^	RO5^b^	Polarizability	Ovality	Area (Å^2^)	Volume (Å^3^)
Azomycin **3**	-	0.21/0.81	0	48.02	1.19	120.7	95.71
**4**	2	0.78/2.12	0	57.8	1.41	245.2	216.3
**5**	3	1.09/2.40	0	59.3	1.45	266.9	234.9
**6**	4	1.40/2.68	0	60.8	1.48	286.1	253.2
**7**	5	1.46/3.10	0	62.3	1.51	306.7	271.6
**8**	6	1.88/3.52	0	63.8	1.54	327.1	290.1
**9**	8	2.36/4.35	0	66.8	1.60	367.9	327.0
**10**	10	2.74/5.19	0	69.8	1.66	408.6	363.9
**11**	12	3.33/6.02	0	72.8	1.71	449.2	400.8

a Predicted with SwissADME Web server/Predicted with Spartan’14 software.

b RO5: number of violations of Lipinski’s rule of 5.

Interestingly, in addition to the increase in connecting points and interactive forces that stabilize the interaction of dimeric compounds with the increasing number of methylene units (Figure 3I–L), it was demonstrated that the 2-nitroimidazole moiety in compounds **10** and **11** can interact more closely than compounds **4**–**9** with the aromatic portion of the FMN structure. This suggests the high ability of the nitro groups in these two dimeric prodrugs to undergo reduction to amino groups. Finally, since the catalytic pocket of TcNTR is external and located at the dimer’s interface, and because the dimeric 2-nitroimidazoles have increased flexibility and capacity to interact with the enzymatic cofactor as methylene units increase, it is likely that not just one but both 2-nitroimidazole moieties of compounds **4**–**11** could be reduced. Compounds **10** and **11** may be more reactive than benznidazole toward *T. cruzi* biomacromolecules.

The frontier molecular orbitals (FMOs) energies, more specifically, the highest occupied molecular orbital (HOMO) and the lowest unoccupied molecular orbital (LUMO), are considered indicators of reactivity between molecules and biological sites, such as cavities of proteins, i.e., compounds with the lowest HOMO–LUMO energy gap can easily suffer chemical reactions.^30,31^ Thus, FMOs values of the dimeric products of 2-nitroimidazoles (**4–11**) and the precursor reagent (**3**) were predicted using Density Functional Theory (DFT) with the method Becke-3-Lee–Yang–Parr (B3LYP) and the standard 6-31G* basis set (see computational details and Figure S1 in the Supporting Information) to comprehend the capacity of designed compounds further to suffer reduction after interaction with TcNTR to support the molecular docking trend. In this sense, the predicted HOMO–LUMO energy gap values for **3**, **4–11** were 4.71, 4.71, 4.70, 4.70, 4.65, 4.68, 4.68, 4.68, and 4.68 eV, respectively, with FMOs density located in the heterocyclic core, where the nitro group can be found, without significant FMOs density in the methylene units (Figure S1 in the Supporting Information). This data reinforces the molecular docking trend that increasing the number of methylene units might improve the chain’s flexibility and its anti-*T. cruzi* profile, i.e., the dimeric compounds **7–11** will present better biological activity than compounds **3–6**. Finally, all planned dimeric compounds have no violations of the Lipinski’s rule of 5 (RO5, Table 1), being considered as potential drugs, as well as the increase in lipophilicity with the rise in the number of methylene units in the homologous series (Log *P*_o/w_, Table 1) will improve the uptake of the designed dimerics to the target to act as anti-*T. cruzi*.

It is important to recognize that the future stability of the obtained molecular docking pose might be evaluated through molecular dynamics simulations over time. A combination of biochemical and biophysical assays, such as determining the enzymatic inhibitory mechanism, performing binding assays to measure interaction strength, and conducting structural experiments, should be carried out to better understand how dimeric 2-nitroimidazoles (**4**–**11**) target TcNTR.^34^ However, in this study, as an initial step to support the *in silico* anti-*T. cruzi* profile of **3**, **4**–**11**, *in vitro* antiparasitic assays were performed after synthesizing the designed dimeric compounds.

### 2.2 Chemistry

First, to validate the *in silico* antiparasitic predictions, the proposed dimeric products of 2-nitroimidazoles (Figure 2) were synthesized via a classical S_N_2 mechanism, which is responsible for the *N*-alkylation. The synthesis was performed using commercial 2-nitro-1*H*-imidazole in excess (0.5 mmol) relative to the dibromoalkane (0.1 mmol) used to produce each product. This process was carried out under heating (50 °C) with dry *N,N*-dimethylformamide (DMF) as the solvent in an inert nitrogen (N_2_) atmosphere and triethylamine (TEA) as the base (0.3 mmol) (Scheme 1).

**Scheme 1:**
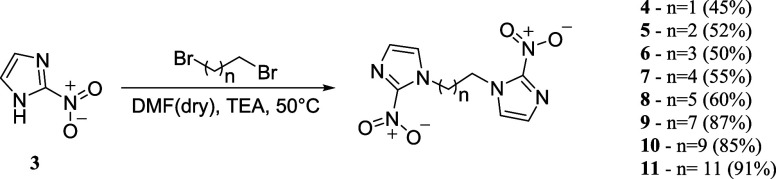
Reaction of 2-Nitroimidazole with Appropriate Dibromoalkanes (Yields of Each Derivative Are Indicated in Brackets)

Reaction times were 24 h, with yields ranging from 45% to 91%. All products (Figure 2) were confirmed by ^1^H and ^13^C NMR and HRMS analysis, and their purity was verified by HPLC (greater than 95%). Thus, the optimized synthetic methodology in this work produced the intended dimers in medium to good yields and under milder reaction conditions than those described by Long and colleagues (1991) for synthesizing dimers **4** and **5**.^34^ Dimer **11** was also described in patent CN106692059 (2017), along with its characterization by ^1^H NMR.^35^

All obtained dimers had their structures confirmed by ^1^H and ^13^C NMR, HRMS, and melting point. In the ^1^H NMR spectrum (Figure S2), a singlet can be observed, integrating for four hydrogens at δ 5.11 ppm, corresponding to the double CH_2_ of the spacer between the pharmacophore group. In the ^13^C NMR (DEPT-135) spectrum (Figure S3), a signal appears at δ 49.55 ppm, indicating two methylene carbons sharing the same signal, bound to the nitrogen atom of the nitroimidazole core. These signals, consistent with the previously highlighted structure of the synthesized compounds, support the thesis that *N*-alkylation was successful. Similar signals can be observed in comparable spectral regions for all eight dimers in the figures, which refer to the NMR spectra in the Supporting Information, confirming their structures and the successful formation of molecular dimers.

### 2.3 Biological Assays

The obtained dimeric compounds **4**–**11** were evaluated for their cytotoxicity profile against mammalian cells, as well as their antiparasitic activity against amastigotes of *T. cruzi* (Tulahuen C2C4 *LacZ* strain) and tested against the bloodstream trypomastigotes of *T. b. brucei*.^34,35^ All molecules exhibited a good cytotoxicity profile against the mammalian cell models tested, including LLC-MK2 fibroblasts, human kidney cells (HEK-293), and mouse macrophages (RAW 264.7) (Tables 2 and 3).

**Table 2:** Anti-*T. Cruzi* Activity of **3** and the Dimeric Compounds **4–11**

Compound	Activity against Amastigotes of *T. cruzi*(Tulahuen C2C4*lacZ*) IC_50_ (μM)	Cytotoxicity in LLC-MK2IC_50_ (μM)	SI^a^ (LLC-MK2)
Azomycin (**3**)	5.74 ± 1.89	>500	>87.10
**4**	9.13 ± 0.93	>200	>21.90
**5**	3.20 ± 0.58	>200	>62.50
**6**	2.66 ± 0.78	>200	>75.18
**7**	1.38 ± 0.24	>200	>144.9
**8**	0.59 ± 0.01	>200	>338.98
**9**	1.19 ± 0.31	>100	>84.03
**10**	0.48 ± 0.11	>100	>208.33
**11**	0.61 ± 0.13	52.39 ± 4.13	85.88
Benznidazole^b^ (**1**)	1.50 ± 0.33	>200	>133.33

a SI* = Selectivity Index = (IC_50_ LLC-MK2)/(IC_50_ *T. cruzi*).

b Reference drug for *T. cruzi*.

**Table 3:** Anti-*T. b. brucei* Activity of **3** and the Dimeric Compounds **4**–**11**

Compounds	*T. b. brucei* 48 h	HEK-293 48 h	RAW-264.7 48 h	SI^a^ (HEK-293)	SI^b^ (RAW-264.7)
IC_50_ (μM)					
**4**	21.42 ± 1.146	>125	>125	>5.83	>5.83
**5**	16.36 ± 2.65	>125	>125	>4.57	>4.57
**6**	24.89 ± 7.89	>125	>125	>5.02	>5.02
**7**	10.06 ± 2.47	>125	>125	>12.42	>12.42
**8**	5.37 ± 3.15	87.41 ± 1.20	>125	16.27	>23.27
**9**	7.98 ± 3.12	22.32 ± 1.08	71.71 ± 3.67	2.79	8.98
**10**	21.04 ± 1.90	35.14 ± 5.31	98.38 ± 9.39	1.67	4.67
**11**	1.79 ± 0.63	102.05 ± 8.54	>125	57.01	>69.83
**Fexinidazole** ^c^ (**2**)	1.20 ± 0.51	-	-	-	-

a SI = Selectivity Index = (IC_50_ HEK-293)/(IC_50_ *T. brucei brucei*).

b SI = Selectivity Index = (IC_50_ RAW-264.7)/(IC_50_ *T. brucei brucei*).

c Reference drug to *T. brucei*.

As previously identified through an *in silico* approach, the antiparasitic activity of the dimeric series shows promise for targeting *T. cruzi*; however, we also evaluated *in vitro* against *T. b. brucei* and obtained a good selective index, demonstrating the potential of the dimeric compounds to be considered as broad-spectrum agents against *Trypanosoma*. We observed an increase in activity against the amastigote forms of *T. cruzi* as the spacer chain length between the 2-nitroimidazole fragments increased, supporting both the molecular docking trend to TcNTR and the theoretical HOMO–LUMO energy gap values, reinforcing that TcNTR is a feasible target for the assayed dimeric compounds. Compounds **8**, **10**, and **11** exhibited half-maximal inhibitory concentration (IC_50_) values in the submicromolar range, outperforming benznidazole, the standard drug for treating *T. cruzi* infections. It is essential to note that compounds with spacers longer than five CH_2_ units in the connection chain exhibited potency similar to that of benznidazole, highlighting compounds 8 and 10, which have a selective index higher than that of benznidazole. This suggests that highly effective anti-*T. cruzi* activity depends on the length of the methylene chain (Table 2), agreeing with the antiparasitic prediction of the improvement of flexibility of the dimeric compounds with the increase of methylene units that might positively impact the capacity of the pharmacophoric core to interact with the enzymatic cofactor, leading to not just one but both 2-nitroimidazole moieties of dimeric compounds to be reduced.

Additionally, the dimers displayed a significant biological profile for anti-*T. b. brucei* activity, demonstrating high selectivity but moderate IC_50_ against the parasite. Only compound **11** showed potency comparable to that of the reference drug, fexinidazole, and a high selectivity index (Table 3). Since we designed eight dimeric 2-nitroimidazoles to target TcNTR by increasing the methylene chain length, this approach did not correlate experimentally with the observed anti-*T. b. brucei* profile, and further molecular docking calculations after *in vitro* assays were not performed on *T. b. brucei* NTR enzyme.

It is important to note that the observed increase in antiparasitic activity may also be attributed to the greater conformational flexibility of the designed structures, which enables the pharmacophoric groups at their ends to interact more freely with the predicted target of the parasite. This increased activity can also be attributed to the theoretical Log *P*_o/w_ value, which enhances their activity through lipophilicity (Table 1). Thus, following the *in silico* predictions in Section 2.1, the effectiveness of these dimers with a longer spacer chain may also be due to their ability to penetrate the parasite’s membrane. Log *P*_o/w_ values were calculated using the method reported by Daina and colleagues (2014) via the SwissADME Web server, supported by Spartan’14 software.^32^

## 3 Materials and Methods

### 3.1 Chemistry

Unless otherwise stated, all chemical reagents were purchased from Sigma–Aldrich (St. Louis, MO, USA). Solvents were treated with activated molecular sieves (3 Å) before use. Reactions were monitored by thin-layer chromatography (TLC) on 0.25 mm Merck (Darmstadt, Germany) silica gel plates (60F-254) and visualized under a UV lamp (254 and 365 nm). All melting points (mp) were uncorrected and measured using a Fisatom 430D apparatus (São Paulo, Brazil) and were uncorrected. ^1^H NMR and ^13^C NMR spectra were recorded on a Bruker Ultrashield Plus spectrometer (Billerica, MA, USA) at 25 °C, referenced to tetramethylsilane (TMS). ^13^C NMR spectra were recorded using DEPT-135 pulse sequences, allowing differentiation of CH, CH_2_ and CH_3_ carbon signals. Chemical shifts were reported in parts per million (ppm, δ) using the residual solvent line as an internal standard. Splitting patterns are designed as s, singlet; d, doublet; t, triplet; m, multiplet; brs, broad singlet. The liquid chromatography–mass spectrometry (LC-MS) analyses were performed on a Shimadzu LC-MS-2020 (Shimadzu Inc., Kyoto, Japan). Analytical conditions: column: Kromasil C18, 150 mm × 4.6 mm × 5 μm (AkzoNobel, Amsterdam, the Netherlands); mobile phase: water with 0.1% formic acid (A), acetonitrile with 0.1% formic acid (B), 1.0 mL/min, linear gradient (indicated on trace); injection volume: 10 μL; detectors: PDA (200–400 nm), ESI+ (low resolution). The liquid high-resolution chromatography–mass spectrometry (LCMS-HR) analyses were performed using an Agilent LC-MS-QTOF 6530C (Agilent Technologies, CA, USA). Analytical conditions: column: Poroshell C18, 100 mm × 4.6 mm × 2.1 μm (Phenomenex, Inc., California, USA); mobile phase: water with 0.1% formic acid (A), acetonitrile with 0.1% formic acid (B), 1.0 mL/min, linear gradient (indicated on trace); injection volume: 4 μL; detectors: PDA (200–400 nm).

#### 3.1.1 General Synthesis Procedure

The suitable dibromoalkane (0.1 mmol), 2-nitroimidazole (**3**) (0.5 mmol equivalent), and TEA (0.3 mmol equivalent) were dissolved in DMF (1 mL) in a round-bottom flask. The reaction mixture was stirred at 60 °C for 24 h. Once the complete consumption of **3** was confirmed by analytical TLC (hexanes:DCM:ethyl acetate, 5:2:3), 20 mL of cold distilled water was added, and the mixture was stirred until a precipitate formed. The crystals were filtered under vacuum, and the product was dried at room temperature.

##### 3.1.1.1 Preparation of 1,2-Bis­(2-nitro-1*H*-imidazol-1-yl)­ethane (**4**)

1,2-Dibromoethane (18.8 mg, 0.1 mmol) was used as the dibromoalkane in this reaction with 2-nitroimidazole (56.5 mg, 0.5 mmol) and TEA (22 μL, 0.3 mmol). The product was isolated as yellow crystals (11.3 mg, 45% yield). Mp = 252–255 °C (Lit.: 240–241 °C).^35^ Structure was confirmed by ^1^H NMR, ^13^C NMR, IR, and MS: ^1^H NMR (500 MHz, Acetone-*d*_6_): δ 7.29 (s, 2H); 7.08 (s, 2H); 5.11 (s, 4H). ^13^C NMR (DEPT-135) (125 MHz, Acetone-*d*_6_, DEPT-135): δ 127.95; 127.19; 49.55. HRMS (EI, *m*/*z*) calculated for C_8_H_9_N_6_O_4_^+^, 253.0685; found 253.0639. HPLC (Gradient; ACN:H_2_O (5–95% ACN), 15 min; 0.1% formic acid): r.t. = 5.37 min, purity 95.77%. All spectra obtained from **4** are available in the (Supporting Information Figures S2–S5).

##### 3.1.1.2 Preparation of 1,3-Bis­(2-nitro-1*H*-imidazol-1-yl)­propane (**5**)

1,3-Dibromopropane (20.18 mg, 0.1 mmol) was used as the dibromoalkane in this reaction with 2-nitroimidazole (56.5 mg, 0.5 mmol) and TEA (22 μL, 0.3 mmol). The product was isolated as yellow crystals (13.8 mg, 52% yield). Mp = 257–260 °C (Lit.: 194–196 °C).^35^ Structure was confirmed by ^1^H NMR, ^13^C NMR, IR, and MS: ^1^H NMR (500 MHz, Acetone-*d*_6_): δ 7.63 (s, 2H); 7.15 (s, 2H); 4.86 (t, *J* = 7.25 Hz, 4H); 2.59 (q, *J* = 7.25 Hz, 2H). ^13^C NMR (125 MHz, Acetone-*d*_6_, DEPT-135): δ 127.92; 126.89; 47.02; 30.80. HRMS (EI, *m*/*z*) calculated for C_9_H_11_N_6_O_4_^+^, 267.0841; found 267.0849. HPLC (Gradient; ACN:H_2_O (5–95% ACN), 15 min; 0.1% formic acid): r.t. = 5.70 min, purity 98.96%. All spectra obtained from **5** are available in the (Supporting Information Figures S6–S9).

##### 3.1.1.3 Preparation of 1,4-Bis­(2-nitro-1*H*-imidazol-1-yl)­butane (**6**)

1,4-Dibromobutane (21.5 mg, 0.1 mmol) was used as the dibromoalkane in this reaction with 2-nitroimidazole (56.5 mg, 0.5 mmol) and TEA (22 μL, 0.3 mmol). The product was isolated as yellow crystals (14 mg, 50% yield). Mp = 217–220 °C. Structure was confirmed by ^1^H NMR, ^13^C NMR, IR, and MS: ^1^H NMR (500 MHz, DMSO-*d*_6_): δ 7.69 (s, 2H); 7.18 (s, 2H); 5.50 (s, 4H); 1.80 (s, 4H). ^13^C NMR (125 MHz, DMSO-*d*_6_, DEPT-135): δ 130.45; 129.23; 122.41; 109.71. HRMS (EI, *m*/*z*) calculated for C_10_H_13_N_6_O_4_^+^, 281.0998; found 281.1007. HPLC (Gradient; ACN:H_2_O (5–95% ACN), 15 min; 0.1% formic acid): r.t. = 6.25 min, purity 99.70%. All spectra obtained from **6** are available in the (Supporting Information Figures S10–S13).

##### 3.1.1.4 Preparation of 1,5-Bis­(2-nitro-1*H*-imidazol-1-yl)­pentane (**7**)

1,5-Dibromopentane (22.9 mg, 0.1 mmol) was used as the dibromoalkane in this reaction with 2-nitroimidazole (56.5 mg, 0.5 mmol) and TEA (22 μL, 0.3 mmol). The product was isolated as yellow crystals (16.2 mg, 55% yield). Mp = 98–101 °C. The structure was confirmed by ^1^H NMR, ^13^C NMR, IR, and MS: ^1^H NMR (500 MHz, DMSO-*d*_6_): δ 7.68 (s, 2H); 7.19 (s, 2H); 4.38 (t, *J* = 7.25 Hz, 4H); 1.81 (q, *J* = 7.41 Hz, 4H); 1.28 (q, *J* = 7.67 Hz, 2H). ^13^C NMR (125 MHz, DMSO-*d*_6_, DEPT-135): δ 128.32; 46.60; 29.62; 23.06. HRMS (EI, *m*/*z*) calculated for C_11_H_15_N_6_O_4_^+^, 295.1154; found 295.1168. HPLC (Gradient; ACN:H_2_O (5–95% ACN), 15 min; 0.1% formic acid): r.t. = 6.72 min, purity 96.74%. All spectra obtained from **7** are available in the (Supporting Information Figures S14–S17).

##### 3.1.1.5 Preparation of 1,6-Bis­(2-nitro-1*H*-imidazol-1-yl)­hexane (**8**)

1,6-Dibromohexane (24.3 mg, 0.1 mmol) was used as the dibromoalkane in this reaction with 2-nitroimidazole (56.5 mg, 0.5 mmol) and TEA (22 μL, 0.3 mmol). The product was isolated as yellow crystals (18.5 mg, 60% yield). Mp = 180–183 °C. Structure was confirmed by ^1^H NMR, ^13^C NMR, IR, and MS: ^1^H NMR (500 MHz, DMSO-*d*_6_): δ 7.68 (s, 2H); 7.18 (s, 2H); 4.36 (t, *J* = 7.6 Hz, 4H); 1.76–1.78 (m, 4H); 1.29–1.32 (m, 4H). ^13^C NMR (125 MHz, DMSO-*d*_6_, DEPT-135): δ 128.31; 49.75; 30.02; 25.73. HRMS (EI, *m*/*z*) calculated for C_12_H_17_N_6_O_4_^+^, 309.1311; found 309.1318. HPLC (Gradient; ACN:H_2_O (5–95% ACN), 15 min; 0.1% formic acid): r.t. = 7.24 min, purity 98.50%. All spectra obtained from **8** are available in the (Supporting Information Figures S18–S21).

##### 3.1.1.6 Preparation of 1,8-Bis­(2-nitro-1*H*-imidazol-1-yl)­octane (**9**)

1,8-Dibromooctane (27.2 mg, 0.1 mmol) was used as the dibromoalkane in this reaction with 2-nitroimidazole (56.5 mg, 0.5 mmol) and TEA (22 μL, 0.3 mmol). The product was isolated as yellow crystals (29.25 mg, 87% yield). Mp = 149–150 °C. Structure was confirmed by ^1^H NMR, ^13^C NMR, IR, and MS: ^1^H NMR (500 MHz, CDCl_3_): δ 7.17 (s, 2H); 7.10 (s, 2H); 4.42 (t, *J* = 7.4 Hz, 4H); 1.85–1.87 (m, 4H); 1.36 (s, *J* = 7.4 Hz, 8H). ^13^C NMR (125 MHz, CDCl_3_, DEPT-135): δ 128.4; 125.8; 50.24; 30.47; 28.71; 26.18. HRMS (EI, *m*/*z*) calculated for C_14_H_21_N_6_O_4_^+^, 337.1624; found 337.1633. HPLC (Gradient; ACN:H_2_O (5–95% ACN), 15 min; 0.1% formic acid): r.t. = 8.29 min, purity 96.76%. All spectra obtained from **9** are available in the (Supporting Information Figures S22–S25).

##### 3.1.1.7 Preparation of 1,10-Bis­(2-nitro-1*H*-imidazol-1-yl)­decane (**10**)

1,10-Dibromodecane (30.0 mg, 0.1 mmol) was used as the dibromoalkane in this reaction with 2-nitroimidazole (56.5 mg, 0.5 mmol) and TEA (22 μL, 0.3 mmol). The product was isolated as yellow crystals (30.9 mg, 85% yield). Mp = 109–110 °C. Structure was confirmed by ^1^H NMR, ^13^C NMR, IR, and MS: ^1^H NMR (500 MHz, CDCl_3_): δ 7.16 (s, 2H); 7.11 (s, 2H); 4.42 (t, *J* = 7.2 Hz, 4H); 1.83–1.88 (m, 4H); 1.28–1.34 (m, 12H). ^13^C NMR (125 MHz, CDCl_3_, DEPT-135): δ 128.36; 125.85; 50.34; 30.50; 29.12; 28.85; 26.27. HRMS (EI, *m*/*z*) calculated for C_16_H_25_N_6_O_4_^+^, 365.1937; found 365.1942. HPLC (Gradient; ACN:H_2_O (5–95% ACN), 15 min; 0.1% formic acid): r.t. = 9.35 min, purity 96.80%. All spectra obtained from **10** are available in the (Supporting Information Figures S26–S29).

##### 3.1.1.8 Preparation of 1,12-Bis­(2-nitro-1*H*-imidazol-1-yl)­dodecane (**11**)

1,12-Dibromododecane (32.8 mg, 0.1 mmol) was used as the dibromoalkane in this reaction with 2-nitroimidazole (56.5 mg, 0.5 mmol) and TEA (22 μL, 0.3 mmol). The product was isolated as yellow crystals (35.6 mg, 91% yield). Mp = 97–100 °C. Structure was confirmed by ^1^H NMR, ^13^C NMR, IR, and MS: ^1^H NMR (500 MHz, CDCl_3_): δ 7.16 (s, 2H); 7.11 (s, 2H); 4.42 (t, *J* = 7.2 Hz, 4H); 1.83–1.88 (m, 4H); 1.27–1.34 (m, 14H).^36^ ^13^C NMR (125 MHz, CDCl_3_, DEPT-135): δ 128.32; 125.83; 50.37; 30.53; 29.31; 29.25; 28.92; 26.32. HRMS (EI, *m*/*z*) calculated for C_18_H_29_N_6_O_4_^+^, 393.2250; found 393.2266. HPLC (Gradient; ACN:H_2_O (5–95% ACN), 15 min; 0.1% formic acid): r.t. = 10.43 min, purity 97.03%. All spectra obtained from **10** are available in the (Supporting Information Figures S30–S33).

### 3.2 Biological Assays

Mammalian lineage cells were used to assess the cytotoxicity of the tested compounds. LLC-MK2, HEK-293, and RAW-264 cells (ATCC) were cultured in Dulbecco’s Modified Eagle’s Medium (DMEM) supplemented with 5% fetal bovine serum (FBS) and incubated at 37 °C in 5% CO_2_, with passages every 4–5 days. Cells were detached from the monolayer using a solution containing 0.25% w/v trypsin and 0.04% EDTA.

The antiparasitic activity assays used amastigotes of the Tulahuen C2C4-*LacZ* strain of *T. cruzi*. Amastigotes and trypomastigotes were cultured by successive reinfections in LLC-MK2 cell monolayers in DMEM supplemented with 5% FBS and incubated at 37 °C with 5% CO_2_. Trypomastigotes were collected from the culture supernatant between days 5 and 10 after infection and separated from nonadhered cells by differential centrifugation.

#### 3.2.1 Evaluation of Cytotoxicity against LLC-MK2 Cells

In a flat 96-well plate, a suspension of 1 × 10^4^ LLC-MK2 cells (ATCC) in DMEM supplemented with 2% FBS was added. Cells were incubated at 37 °C (5% CO_2_) for 20 h, then washed with phosphate-buffered saline (PBS) to remove nonadherent cells. Cells were treated with serial dilutions (200–2 μM) of compounds in triplicate, prediluted in DMEM with 2% FBS. Untreated, vehicle (0.2% v/v DMSO), and blank (no cells added) controls were included. After 120 h of incubation, the supernatant was removed, the cell monolayer washed with PBS, and the culture medium refreshed. Then, 20 μL of a 3.0 mM MTT salt solution was added, followed by two additional hours of incubation. The supernatant was removed again, and the MTT formazan crystals were dissolved by adding 120 μL of DMSO per well. After 1.5 h of dark incubation at 37 °C to dissolve the crystals, the absorbance was measured at 570 nm using a plate reader.

#### 3.2.2 Evaluation of Cytotoxicity against HEK-293 Cells

In a 96-well transparent plate, a suspension of 1 × 10^4^ HEK-293 cells (ATCC) in DMEM medium supplemented with 10% FBS was added. Cells were incubated at 37 °C (5% CO_2_) for 20 h and then washed with PBS to remove nonadherent cells. The cells were treated with serial dilutions of the hybrids in triplicate (125–0.2 μM), prediluted in DMEM containing 10% FBS. The experiment included untreated controls, vehicle (0.2% v/v DMSO), and a blank (no added cells). After 120 h of incubation, the supernatant was removed, the cell monolayer was washed with PBS, and the culture medium was replaced. Then, 20 μL of 3.0 mM MTT saline was added, followed by an additional 1.5 h of incubation. The supernatant was removed, and the MTT formazan crystals were dissolved by adding 120 μL of DMSO per well. After incubating for 1.5 h in the dark at 37 °C, the absorbance was measured at 570 nm using a plate reader.

#### 3.2.3 Evaluation of Cytotoxicity against RAW-264.7 Cells

In a 96-well transparent plate, a suspension of 1 × 10^4^ RAW-264.7 cells (ATCC) in Roswell Park Memorial Institute (RPMI) medium supplemented with 10% FBS was added. Cells were incubated at 37 °C (5% CO_2_) for 20 h, then washed with PBS to remove nonadherent cells. Cells were treated with serial dilutions of the hybrids in triplicate (125–0.2 μM), prediluted in RPMI supplemented with 10% FBS. Controls included untreated samples, vehicle (0.2% v/v DMSO), and blank wells (no cells). After 120 h of incubation, the supernatant was removed, the cell monolayer was washed with PBS, and the culture medium was renewed. Then, 20 μL of 3.0 mM MTT saline was added, followed by an additional 1.5 h of incubation. The supernatant was removed again, and the MTT formazan crystals were dissolved by adding 120 μL of DMSO per well. After incubating for 1.5 h in the dark at 37 °C to dissolve the crystals, absorbance was measured at 570 nm using a plate reader.

#### 3.2.4 Evaluation of Trypanocidal Activity against *T. cruzi* Amastigotes

In a flat 96-well plate, a suspension of 1 × 10^4^ LLC-MK2 cells (ATCC) in DMEM supplemented with 2% FBS was added. Cells were incubated at 37 °C with 5% CO_2_ for 4 h to promote adhesion, then washed with PBS to remove nonadherent cells. A suspension containing 1.5 × 10^5^ *T. cruzi* trypomastigote forms of the Tulahuen C2C4 *LacZ* strain was added to the cells, followed by incubation at 37 °C with 5% CO_2_ for 20 h to establish infection. Noninternalized parasites were removed with three successive PBS washes, then treatment with serial dilutions (50–0.08 μM) of compounds in triplicate, prediluted in DMEM with 2% FBS. Controls included untreated cells, vehicle (0.2% v/v DMSO), and blank (no parasite added). Benznidazole, in serial dilutions, served as a positive control. After 5 days (120 h), 30 μL of a 0.5 mM CPRG solution in PBS was added along with 0.9% v/v Igepal CA-630. After a 2h incubation, absorbance was measured at 570 nm using a plate reader.

#### 3.2.5 Evaluation of Trypanocidal Activity against *T. brucei brucei* Bloodstream

In a transparent 96-well plate, the parasites were incubated for 48 h at a concentration of 5 × 10^4^ parasites per well, for 24 h, a concentration of 10 × 10^5^ parasites per well was used in Hirumi’s Modified Iscove’s (HMI-9) medium supplemented with 10% FBS and a final 100 μL/well volume. Treatment was carried out using serial dilutions of the compounds (100–0.02 μM) and fexinidazole as the reference drug, both in triplicate. Triplicates of untreated parasites (live control), parasites treated with DMSO 0.2% v/v (vehicle), and wells without parasites (blank control) served as experimental controls. After incubation, 20 μL of MTS solution (5% PMS) was added, and the sample was incubated for another 2 h. Once the plate was visibly stained—due to the soluble formazan salt produced by the action of viable parasites from the reduction of MTS—the absorbance was measured at 490 nm using a plate reader.

#### 3.2.6 Molecular Docking Procedure

The homodimeric tridimensional structure of TcNTR was predicted using the web server Swiss-model,^37^ using as a template the X-ray nitroreductase structure from *E. coli* B obtained in the Protein Data Bank with access code 1DS7^25^, following the protocol previously described by Cirqueira and colleagues,^26^ which was revalidated with the reported AlphaFold predicted model (ID: AF-Q4DCW9-F1) and with the reported Swiss-Model TcNTR of CL Brener (ID: Q4DCW9_78-311:1nox.1.B). The chemical structure of **3** and the dimeric products of 2-nitroimidazoles **4–11** were built and minimized in terms of energy by Density Functional Theory (DFT) under B3LYP/6-31G* with Spartan’14 software (Wavefunction, Inc., Irvine, CA, USA).^38^ The same method was used to obtain the physicochemical properties of the compounds with Spartan’14 software (Wavefunction, Inc., Irvine, CA, USA).

The molecular docking calculations were performed using the GOLD 2025 software (Cambridge Crystallographic Data Center, Cambridge, CB2 1EZ, UK),^27^ considering a pH of 7.4. Redocking calculations were performed with the cofactor FMN crystallized in the NTR structure from *E. coli* B, yielding root-mean-square deviation (RMSD) values of 1.0529, 0.9275, 1.1619, and 1.4489 Å for ChemPLP, GoldScore, ChemScore, and ASP, respectively. Since ChemPLP yielded an RMSD close to unity, the default function in the GOLD 2025 software was used for the molecular docking calculations, with a 6 Å radius around the cofactor FMN. The web server Protein–Ligand Interaction Profiler (PLIP) was used for the identification of protein–ligand interactions,^39^ and the figures of the docking poses for the largest docking score value were generated with PyMOL Molecular Graphics System 1.0 level software (Delano Scientific LLC software, Schrödinger, New York, NY, USA).^40^

## 4 Conclusions

Designing the dimeric series based on the benznidazole (2-nitroimidazole) pharmacophore was identified as a feasible approach to obtain novel antiparasitic compounds. Molecular docking calculations using the predicted TcNTR structure combined with theoretical physicochemical properties was the first step to the design of the dimers. The synthesis process demonstrated that the dimers could be prepared straightforwardly in a single step via an *N*-alkylation reaction using bidentate alkylating agents, yielding eight dimers, five of which are novel. All compounds were active against the replicative forms of *T. cruzi* (Tulahuen C2C4-*LacZ*), particularly the longer-chain dimers, which exhibited IC_50_ values below 1.0 μM. The *in silico* calculations suggested that the nitroimidazole group might be activated by TcNTR, depending on the length of the compounds’ methylene chain, due to stronger pharmacophore interactions with the enzyme’s active site in longer structures. This activation generates toxic reduction products that can irreversibly damage biological macromolecules, compromising the parasite’s integrity. In addition to being harmful to *T. cruzi* and moderately toxic to *T. b. brucei*, the new derivatives obtained in this study showed very low cytotoxicity against mammalian cells, demonstrating their high selectivity—especially for the longer-chain dimers. The results above indicated the perspective of the most active compound to be further assessed through additional cytotoxicity tests on primary mammalian cells and in an *in vivo* infection model.

## Supplementary Material



## Notes

The frontier molecular orbital density and the corresponding HOMO–LUMO energy gap for the compounds **3–11**, calculated with DFT/B3LYP/6-31G* (Figure S1); ^1^H NMR of **4** in acetone-*d*_6_ (Figure S2); ^13^C NMR DEPT-135 of **4** in acetone-*d*_6_ (Figure S3); HRMS-TOF (MS+) of **4** (Figure S4); HPLC chromatogram of **4** (Figure S5); ^1^H NMR of **5** in acetone-*d*_6_ (Figure S6); ^13^C NMR DEPT-135 of **5** in acetone-*d*_6_ (Figure S7); HRMS-TOF (MS+) of **5** (Figure S8); HPLC chromatogram of **5** (Figure S9); ^1^H NMR of **6** in DMSO-*d*_6_ (Figure S10); ^13^C NMR DEPT-135 of **6** in DMSO-*d*_6_ (Figure S11); HRMS-TOF (MS+) of **6** (Figure S12); HPLC chromatogram of **6** (Figure S13); ^1^H NMR of **7** in DMSO-*d*_6_ (Figure S14); ^13^C NMR DEPT-135 of **7** in DMSO-*d*_6_ (Figure S15); HRMS-TOF (MS+) of **7** (Figure S16); HPLC chromatogram of **7** (Figure S17); ^1^H NMR of **8** in DMSO-*d*_6_ (Figure S18); ^13^C NMR DEPT-135 of **8** in DMSO-*d*_6_ (Figure S19); HRMS-TOF (MS+) of **8** (Figure S20); HPLC chromatogram of **8** (Figure S21); ^1^H NMR of **9** in CDCl_3_ (Figure S22); ^13^C NMR DEPT-135 of **9** in CDCl_3_ (Figure S23); HRMS-TOF (MS+) of **9** (Figure S24); HPLC chromatogram of **9** (Figure S25); ^1^H NMR of **10** in CDCl_3_ (Figure S26); ^13^C NMR DEPT-135 of **10** in CDCl_3_ (Figure S27); HRMS-TOF (MS+) of **10** (Figure S28); HPLC chromatogram of **10** (Figure S29); ^1^H NMR of **11** in CDCl_3_ (Figure S30); ^13^C NMR DEPT-135 of **11** in CDCl_3_ (Figure S31); HRMS-TOF (MS+) of **11** (Figure S32); HPLC chromatogram of **11** (Figure S33); computational details for the structural-energy minimization of compound **3**; computational details for the structural-energy minimization of compound **4**; computational details for the structural-energy minimization of compound **5**; computational details for the structural-energy minimization of compound **6**; computational details for the structural-energy minimization of compound **7**; computational details for the structural-energy minimization of compound **8**; computational details for the structural-energy minimization of compound **9**; computational details for the structural-energy minimization of compound **10;** computational details for the structural-energy minimization of compound **11**. (PDF)
