# Supplementary material for: Contribution to the Chemotherapy of Human Trypanosomiasis: Design, Synthesis, and Biological Evaluation of Dimeric 2‑Nitroimidazoles against Trypanosoma cruzi Amastigotes and Bloodstream Trypanosoma brucei
Source: ACS Omega. 2025 Dec 26;11(1):1546–56. doi: 10.1021/acsomega.5c09284 (PMC12809571; doi:10.1021/acsomega.5c09284)
Supplement: Supplementary file 1 [file ao5c09284_si_001.pdf]

Contribution to the Chemotherapy of Human Trypanosomiasis: Design, Synthesis, and Biological Evaluation of Dimeric 2-Nitroimidazoles Against *Trypanosoma cruzi* Amastigotes and Bloodstream *Trypanosoma brucei*

Afonso Santine M. M. Velez[1], Otávio Augusto Chaves[2,3], Carlos Serpa,[2] Fatma M. Salem[4], Bibo Li[5], Bin Su[4], Célio Geraldo Freire-de-Lima[6], Débora Decoté-Ricardo[7*], Marco Edilson Freire de Lima[1*]

[1] Programa de Pós-graduação em Química, Instituto de Química, Universidade Federal Rural do Rio de Janeiro, Seropédica 23.897-000, RJ, Brazil.

[2] Department of Chemistry, Coimbra Chemistry Center - Institute of Molecular Science (CQC-IMS), University of Coimbra, Rua Larga, 3004-535 Coimbra, Portugal.

[3] Laboratory of Immunopharmacology, Centro de Pesquisa, Inovação e Vigilância em COVID-19 e Emergências Sanitárias (CPIV), Oswaldo Cruz Institute (IOC), Rio de Janeiro 21040-361, Rio de Janeiro, RJ, Brazil.

[4] Department of Chemistry, College of Sciences and Health Professions, Cleveland State University, 2121 Euclid Avenue, Cleveland, Ohio, 44115, United States of America.

[5] Department of Biology, Geo. & Env. Sciences, College of Sciences and Health Professions, Cleveland State University, 2121 Euclid Avenue, Cleveland, Ohio, 44115, United States of America.

[6] Instituto de Biofísica Carlos Chagas Filho, Universidade Federal do Rio de Janeiro, Rio de Janeiro, 21.941-902, RJ, Brazil.

[7] Departamento de Microbiologia e Imunologia Veterinária, Instituto de Veterinária - Universidade Federal Rural do Rio de Janeiro, Seropédica, RJ, Brazil.

Corresponding authors: M.E.F.L. (marcoedilson@gmail.com); D.D.-R.(decotericardo@ufrrj.br).

INDEX

Figure S1. The frontier molecular orbital density and the corresponding HOMO-LUMO energy gap for the compounds **3-11**, calculated with DFT/B3LYP/6-31G*.

Figure S2. ^{1}H-NMR of **4** in Acetone-d_6.

Figure S3. ^{13}C-NMR DEPT-135 of **4** in Acetone-d_6.

Figure S4. HRMS-TOF (MS+) of **4**.

Figure S5. HPLC Chromatogram of **4**.

Figure S6. ^{1}H-NMR of **5** in Acetone-d_6.

Figure S7. ^{13}C-NMR DEPT-135 of **5** in Acetone-d_6.

Figure S8. HRMS-TOF (MS+) of **5**.

Figure S9. HPLC Chromatogram of **5**.

Figure S10. ^{1}H-NMR of **6** in DMSO-d_6.

Figure S11. ^{13}C-NMR DEPT-135 of **6** in DMSO-d_6.

Figure S12. HRMS-TOF (MS+) of **6**.

Figure S13. HPLC Chromatogram of **6**.

Figure S14. ^{1}H-NMR of **7** in DMSO-d_6.

Figure S15. ^{13}C-NMR DEPT-135 of **7** in DMSO-d_6.

Figure S16. HRMS-TOF (MS+) of **7**.

Figure S17. HPLC Chromatogram of **7**.

Figure S18. ^{1}H-NMR of **8** in DMSO-d_6.

Figure S19. ^{13}C-NMR DEPT-135 of **8** in DMSO-d_6.

Figure S20. HRMS-TOF (MS+) of **8**.

Figure S21. HPLC Chromatogram of **8**.

Figure S22. ^{1}H-NMR of **9** in $CDCl_3$.

Figure S23. ^{13}C-NMR DEPT-135 of **9** in $CDCl_3$.

Figure S24. HRMS-TOF (MS+) of **9**.

Figure S25. HPLC Chromatogram of **9**.

Figure S26. ^{1}H-NMR of **10** in $CDCl_3$.

Figure S27. ^{13}C-NMR DEPT-135 of **10** in $CDCl_3$.

Figure S28. HRMS-TOF (MS+) of **10**.

Figure S29. HPLC Chromatogram of **10**.

Figure S30. ^{1}H-NMR of **11** in $CDCl_3$.

Figure S31. ^{13}C-NMR DEPT-135 of **11** in $CDCl_3$.

Figure S32. HRMS-TOF (MS+) of **11**.

Figure S33. HPLC Chromatogram of **11**.

Computational details for the structural-energy minimization of compound 3

Computational details for the structural-energy minimization of compound 4

Computational details for the structural-energy minimization of compound 5

Computational details for the structural-energy minimization of compound 6

Computational details for the structural-energy minimization of compound 7

Computational details for the structural-energy minimization of compound 8

Computational details for the structural-energy minimization of compound 9

Computational details for the structural-energy minimization of compound 10

Computational details for the structural-energy minimization of compound 11

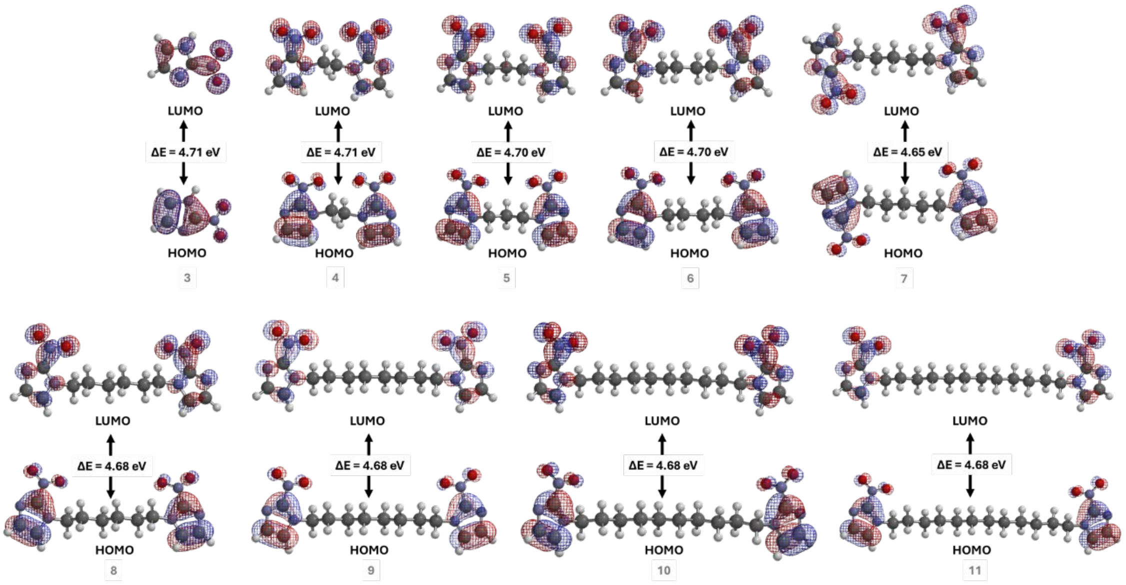

Figure S1. The frontier molecular orbital density and the corresponding HOMO-LUMO energy gap for the compounds **3-11**, calculated with DFT/B3LYP/6-31G*.

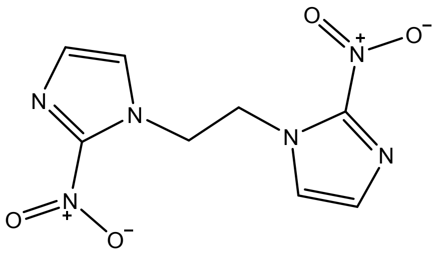

1,2-bis(2-nitro-1*H*-imidazol-1-yl)ethane
Chemical Formula: $C_8H_8N_6O_4$
Exact Mass: 252,06
Molecular Weight: 252,19
m/z: 252.06 (100.0%), 253.06 (8.7%), 253.06 (1.8%)
Elemental Analysis: C, 38.10; H, 3.20; N, 33.32; O, 25.38

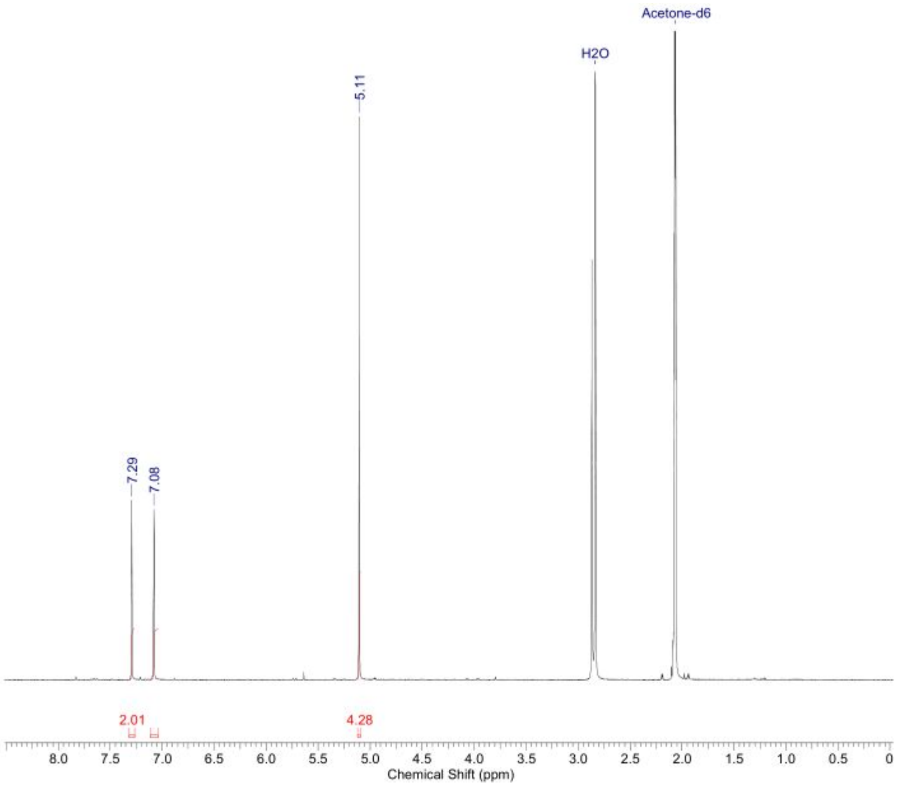

Figure S2. ^{1}H-NMR of **4** in Acetone-d_6.

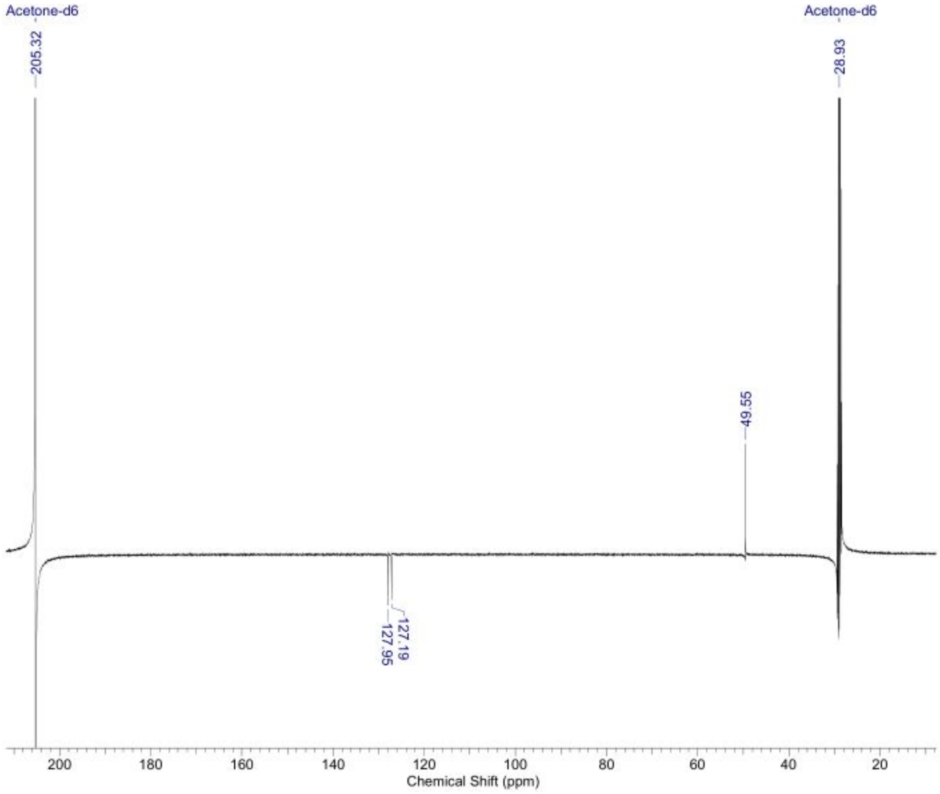

Figure S3. ^{13}C-NMR DEPT-135 of **4** in Acetone-d_6.

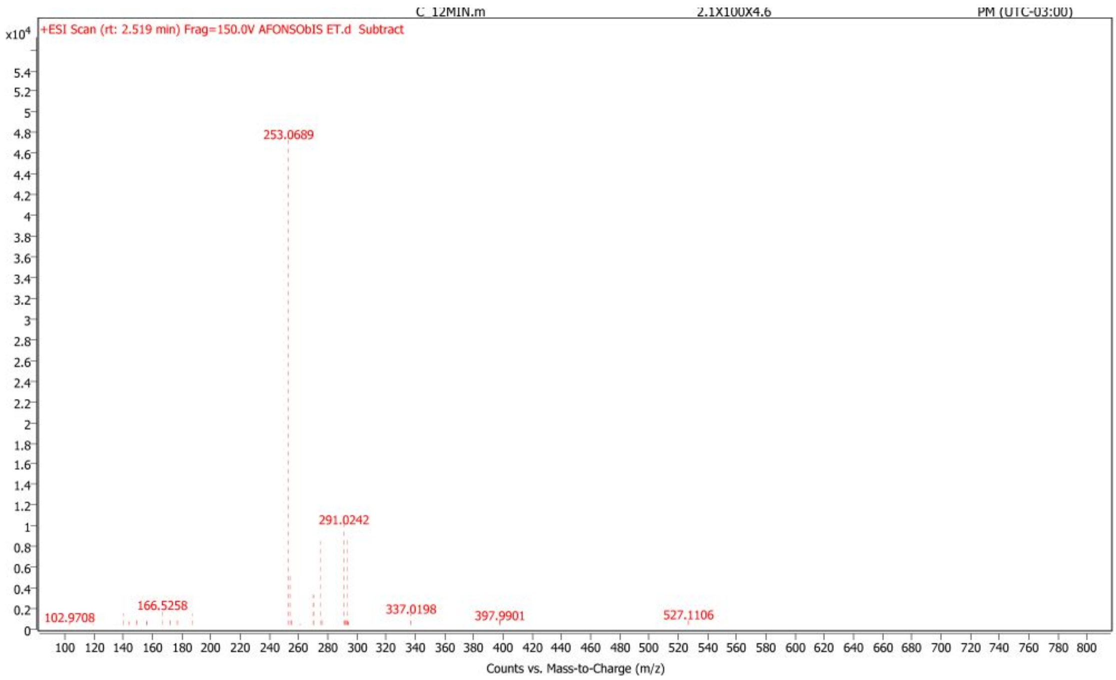

Figure S4. HRMS-TOF (MS+) of **4**.

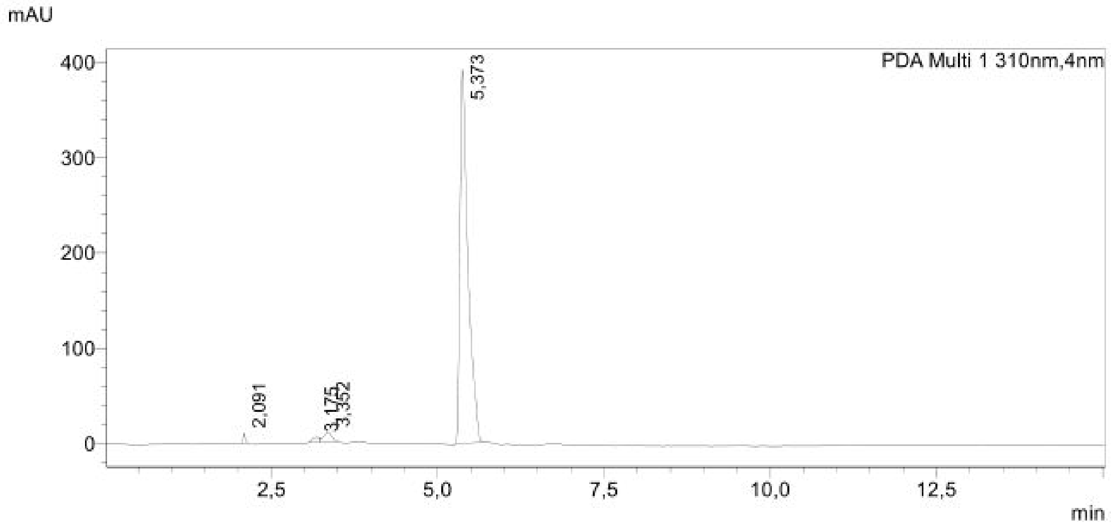

Peak Table

PDA Ch1 310nm

Peak#	Ret. Time	Area	Area%
1	2,091	23815	0,703
2	3,175	37394	1,103
3	3,352	81910	2,417
4	5,373	3246094	95,777
Total		3389213	100,000

Figure S5. HPLC Chromatogram of **4**.

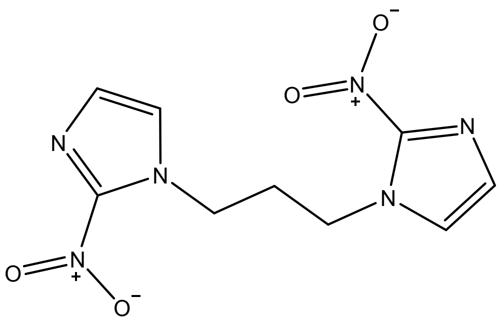

1,3-bis(2-nitro-1*H*-imidazol-1-yl)propane
Chemical Formula: $C_9H_{10}N_6O_4$
Exact Mass: 266,08
Molecular Weight: 266,22
m/z: 266.08 (100.0%), 267.08 (9.7%), 267.07 (2.2%)
Elemental Analysis: C, 40.61; H, 3.79; N, 31.57; O, 24.04

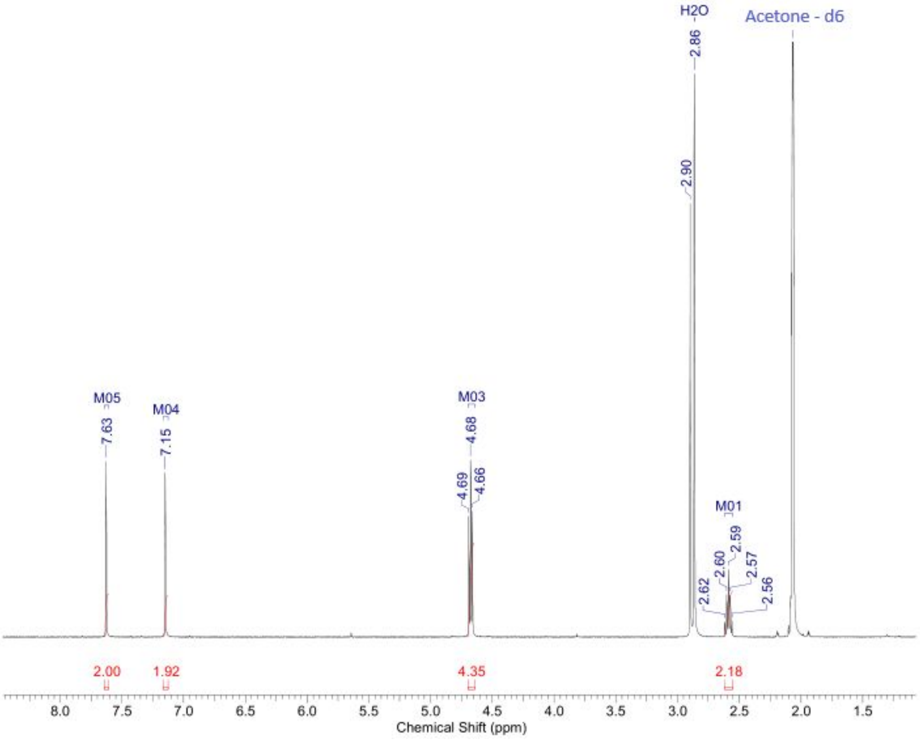

Figure S6. ^{1}H-NMR of **5** in Acetone-d_6.

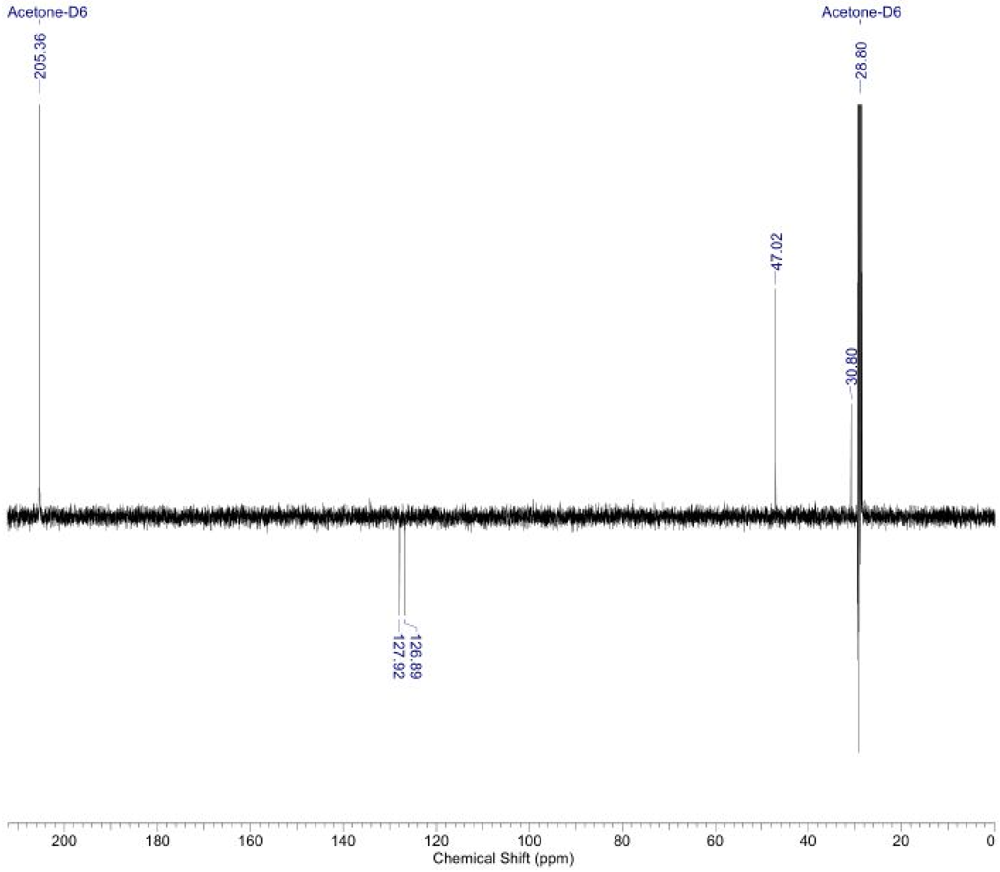

Figure S7. ¹³C-NMR DEPT-135 of **5** in Acetone-d_6.

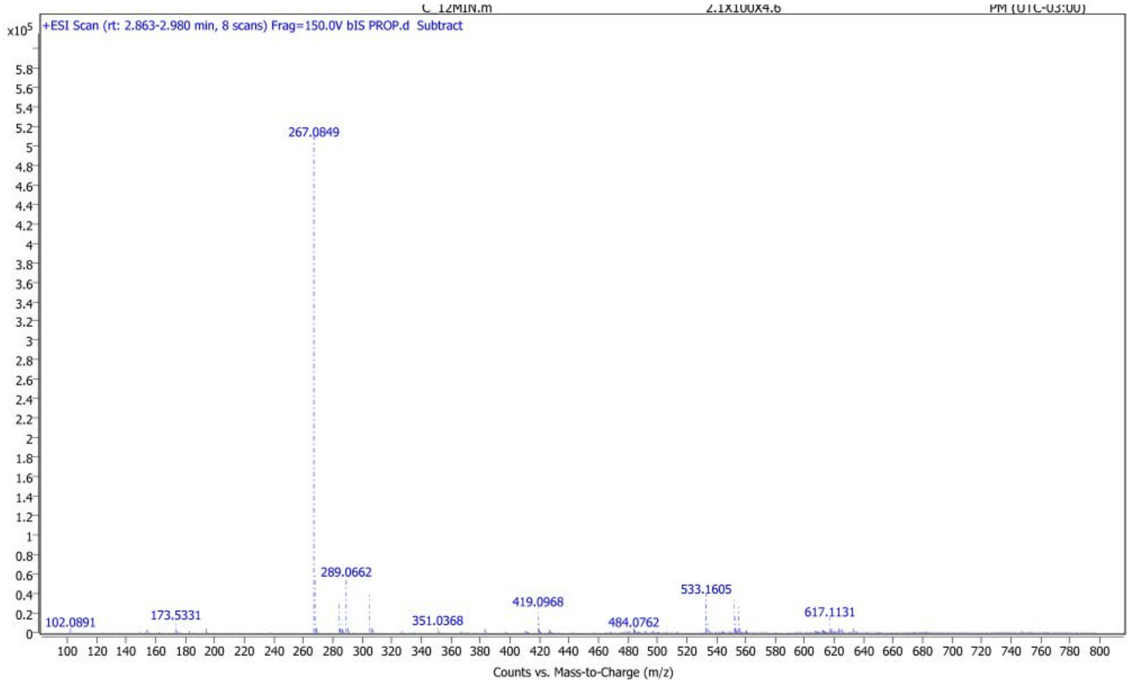

Figure S8. HRMS-TOF (MS+) of **5**.

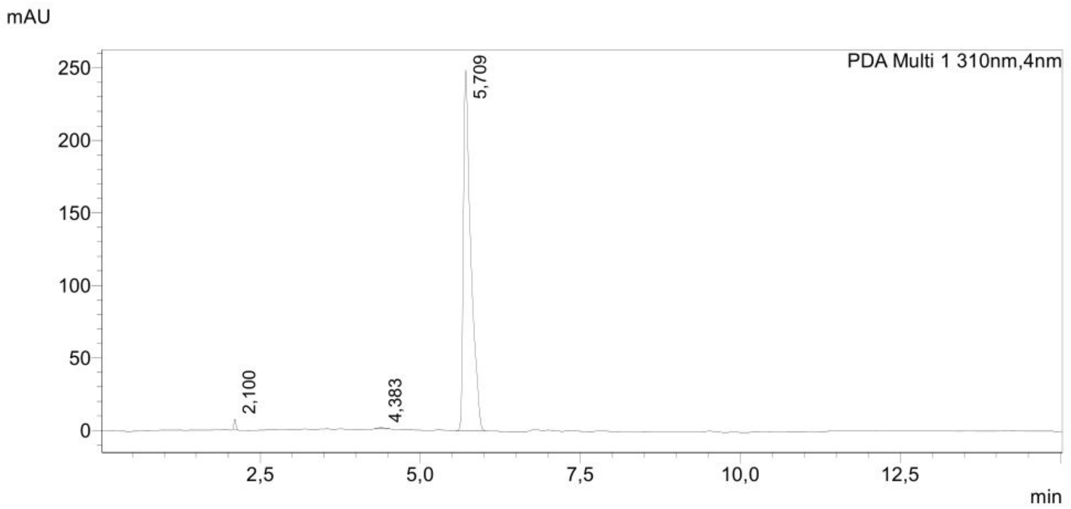

Peak Table

PDA Ch1 310nm

Peak#	Ret. Time	Area	Area%
1	2,100	14929	0,730
2	4,383	6296	0,308
3	5,709	2022725	98,962
Total		2043950	100,000

Figure S9. HPLC Chromatogram of **5**.

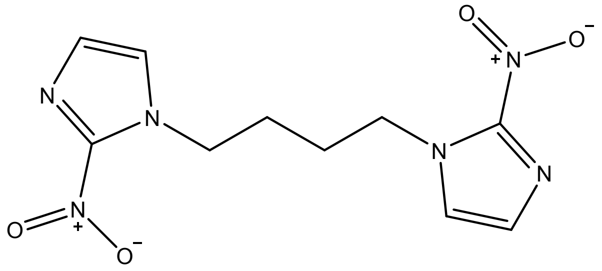

1,4-bis(2-nitro-1*H*-imidazol-1-yl)butane
Chemical Formula: $C_{10}H_{12}N_6O_4$
Exact Mass: 280,09
Molecular Weight: 280,24
m/z: 280.09 (100.0%), 281.10 (10.8%), 281.09 (2.2%)
Elemental Analysis: C, 42.86; H, 4.32; N, 29.99; O, 22.84

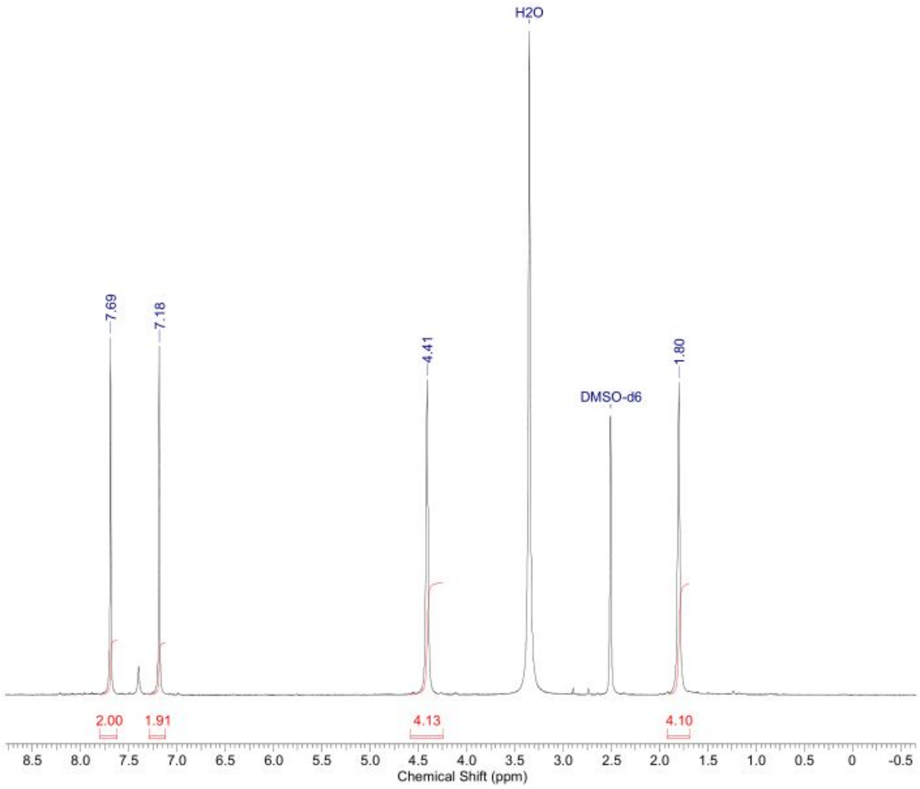

Figure S10. ^{1}H-NMR of **6** in DMSO-d_6.

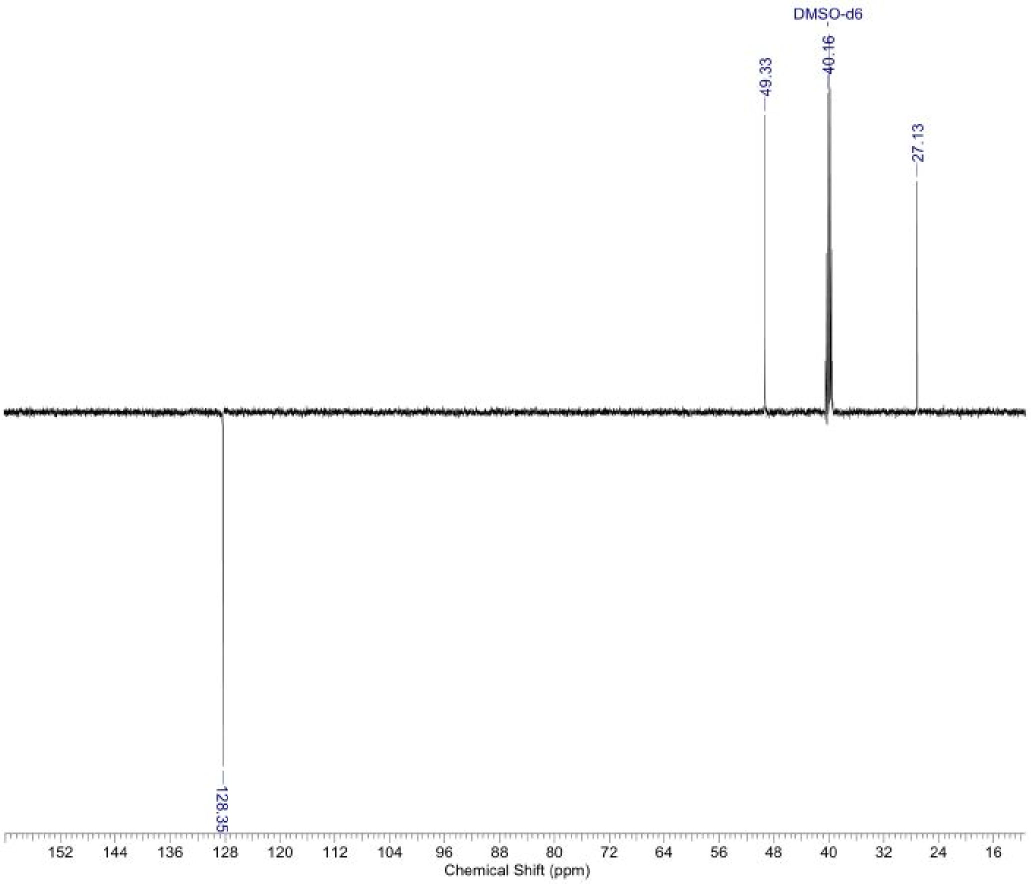

Figure S11. ^{13}C-NMR DEPT-135 of **6** in DMSO-d_6.

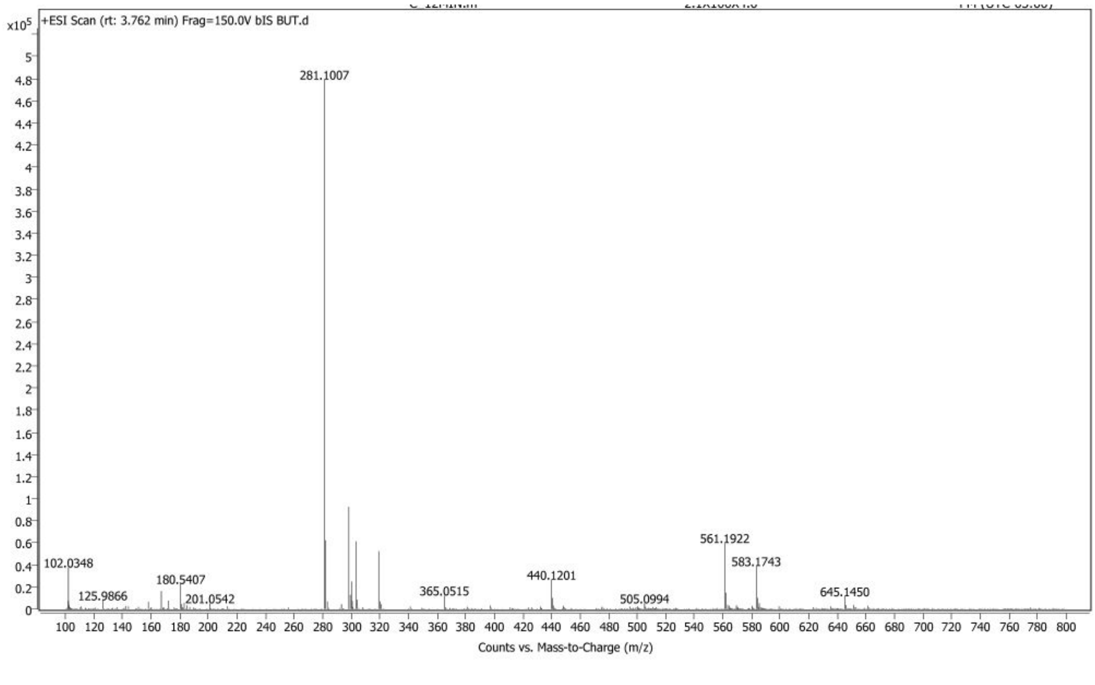

Figure S12. HRMS-TOF (MS+) of **6**.

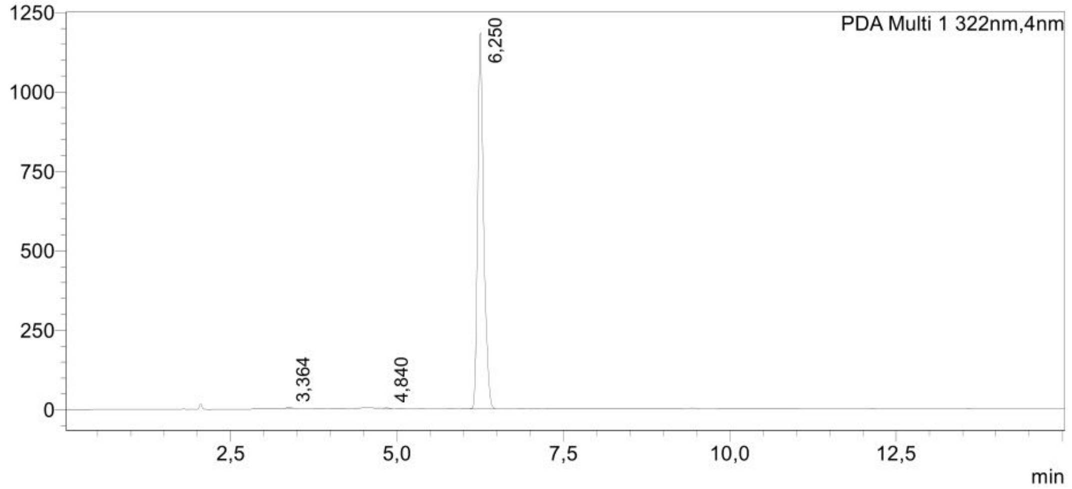

Peak Table

PDA Ch1 322nm

Peak#	Ret. Time	Area	Height	Height%	Area%
1	3,364	12299	2560	0,216	0,164
2	4,840	3925	1103	0,093	0,052
3	6,250	7481721	1182610	99,691	99,784
Total		7497946	1186274	100,000	100,000

Figure S13. HPLC Chromatogram of **6**.

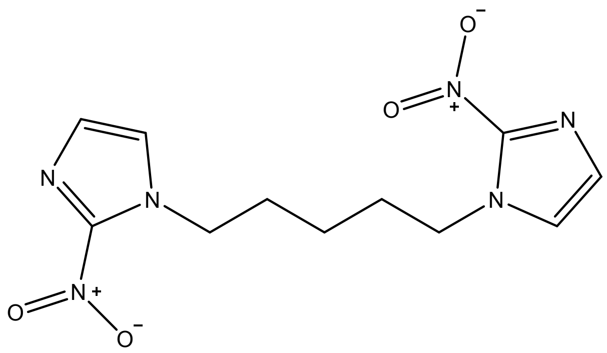

1,5-bis(2-nitro-1*H*-imidazol-1-yl)pentane
Chemical Formula: $C_{11}H_{14}N_6O_4$
Exact Mass: 294,11
Molecular Weight: 294,27
m/z: 294.11 (100.0%), 295.11 (11.9%), 295.10 (2.2%)
Elemental Analysis: C, 44.90; H, 4.80; N, 28.56; O, 21.75

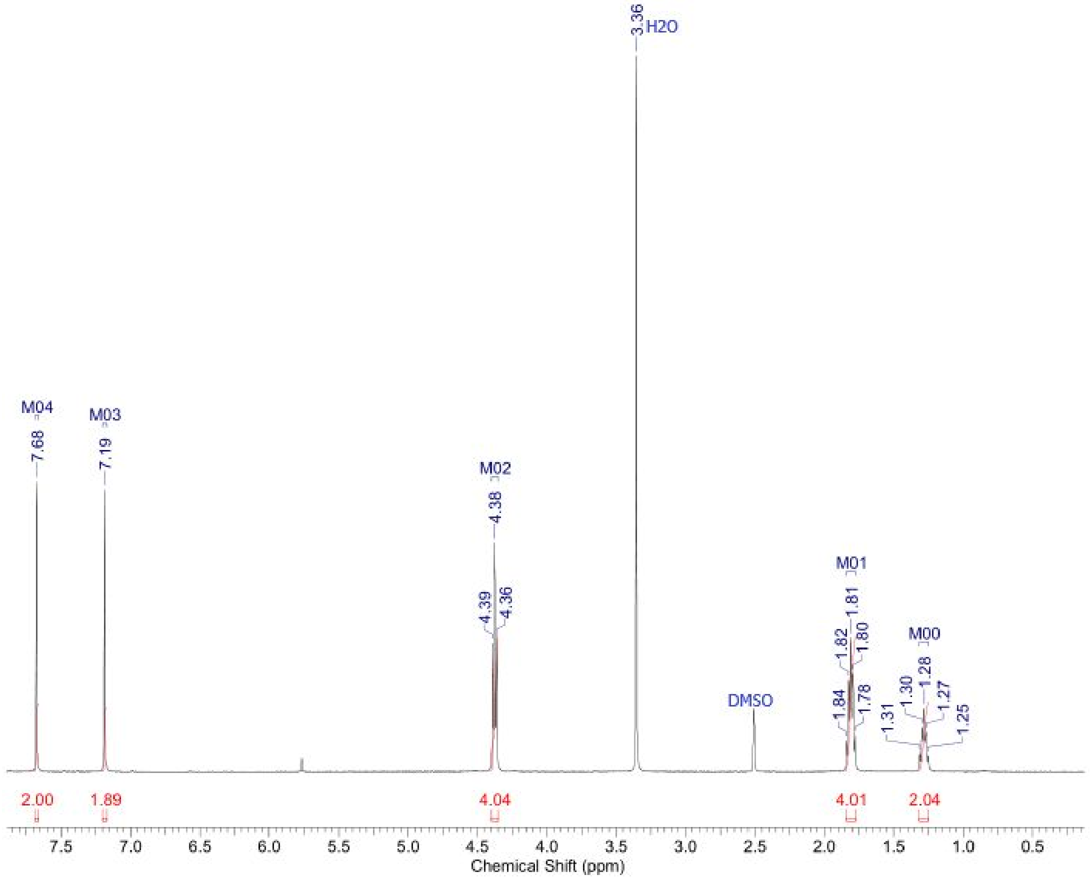

Figure S14. ^{1}H-NMR of **7** in DMSO-d_6.

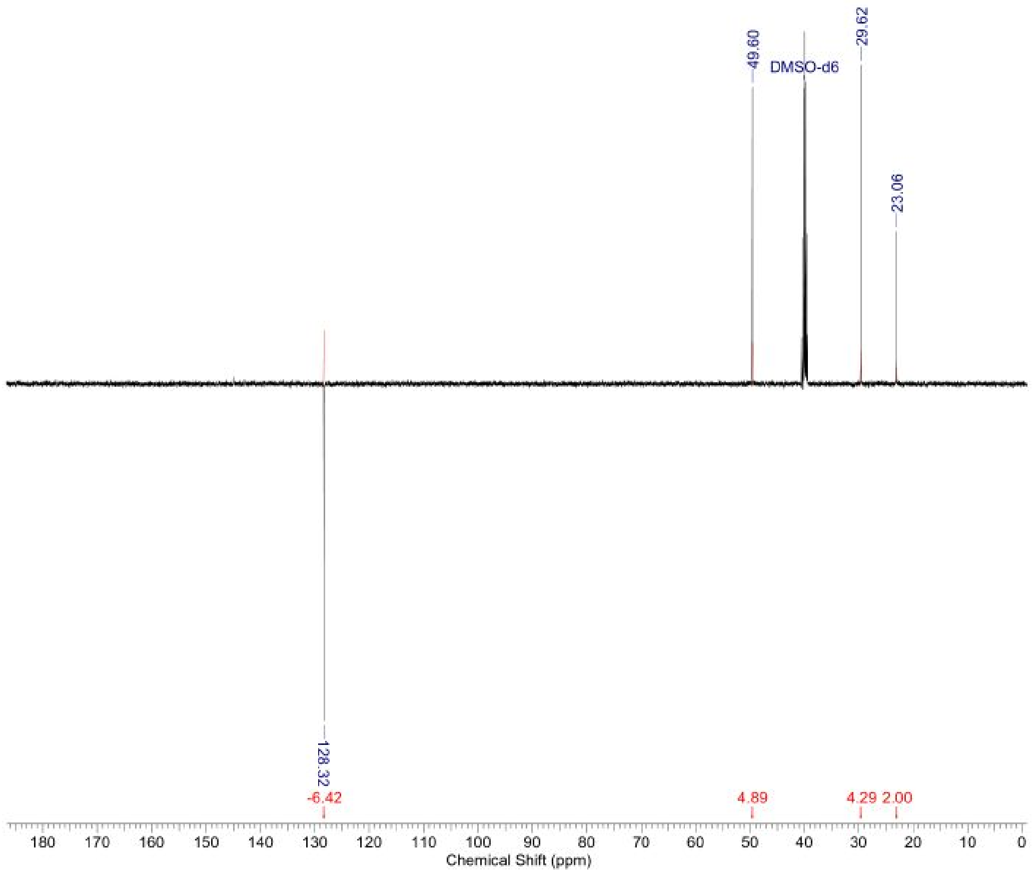

Figure S15. ^{13}C-NMR DEPT-135 of **7** in DMSO-d_6.

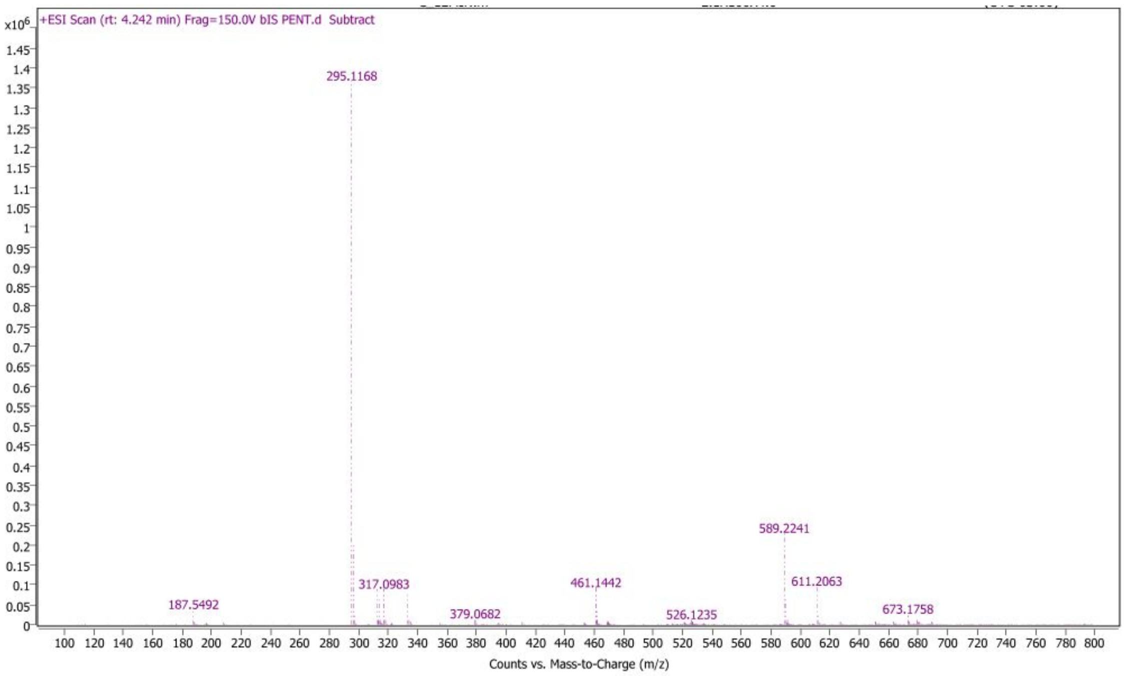

Figure S16. HRMS-TOF (MS+) of **7**.

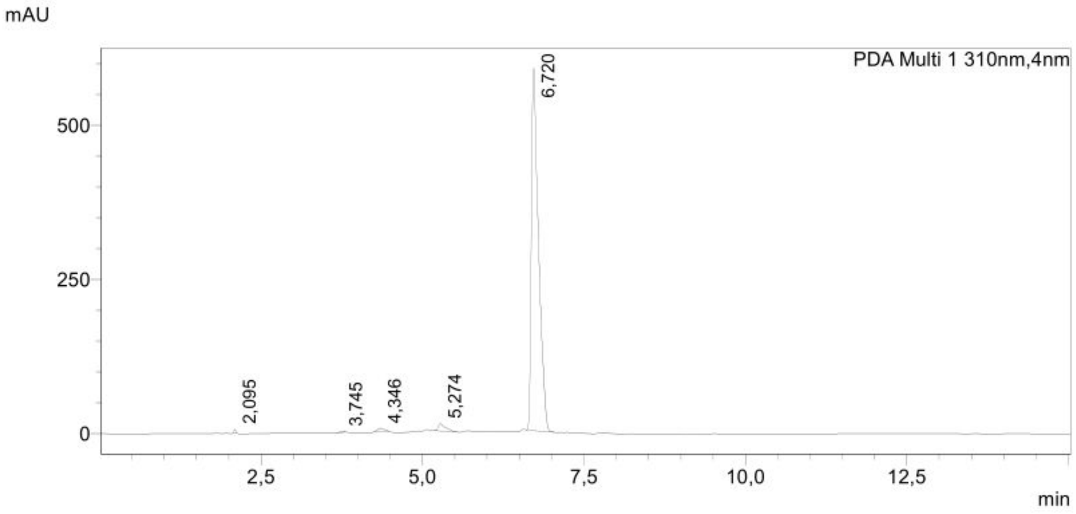

Peak Table

PDA Ch1 310nm

Peak#	Ret. Time	Area	Area%
1	2,095	9917	0,211
2	3,745	7915	0,169
3	4,346	40308	0,858
4	5,274	94975	2,022
5	6,720	4543694	96,740
Total		4696809	100,000

Figure S17. HPLC Chromatogram of **7**.

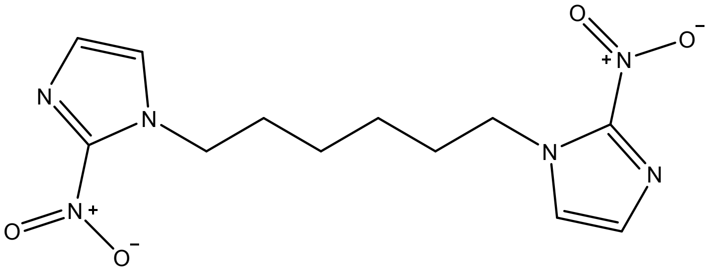

1,6-bis(2-nitro-1*H*-imidazol-1-yl)hexane
Chemical Formula: $C_{12}H_{16}N_6O_4$
Exact Mass: 308,12
Molecular Weight: 308,30
m/z: 308.12 (100.0%), 309.13 (13.0%), 309.12 (2.2%)
Elemental Analysis: C, 46.75; H, 5.23; N, 27.26; O, 20.76

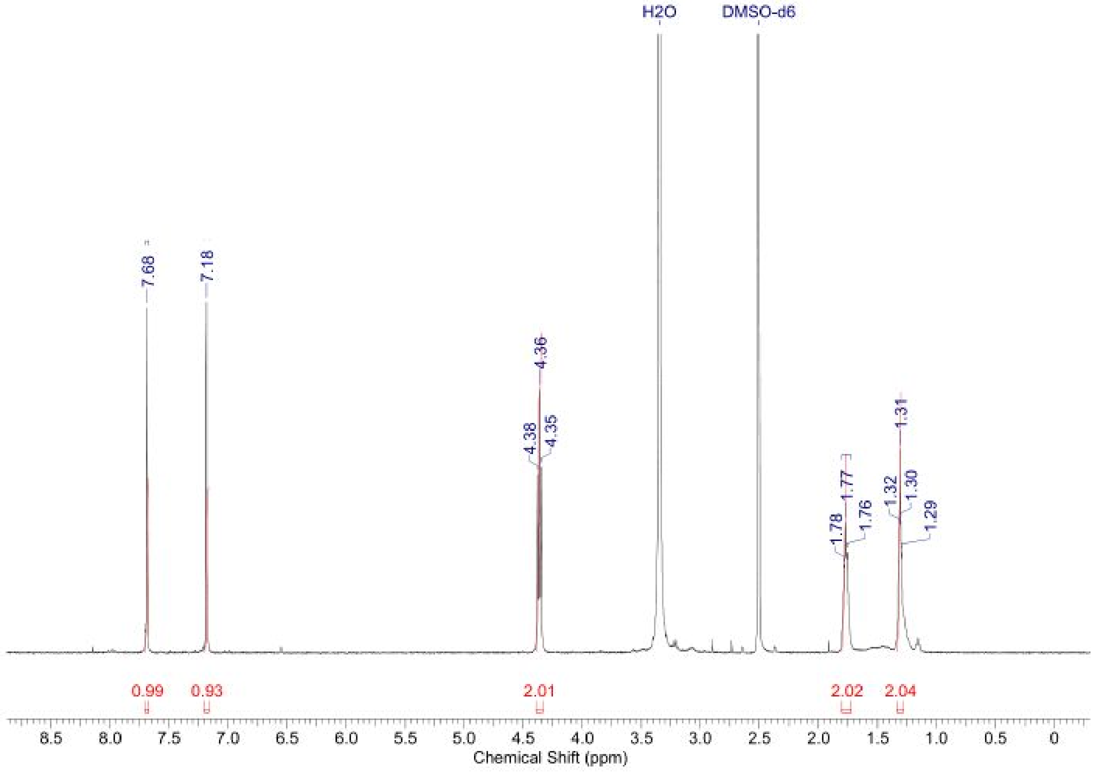

Figure S18. ^{1}H-NMR of **8** in DMSO-d_6.

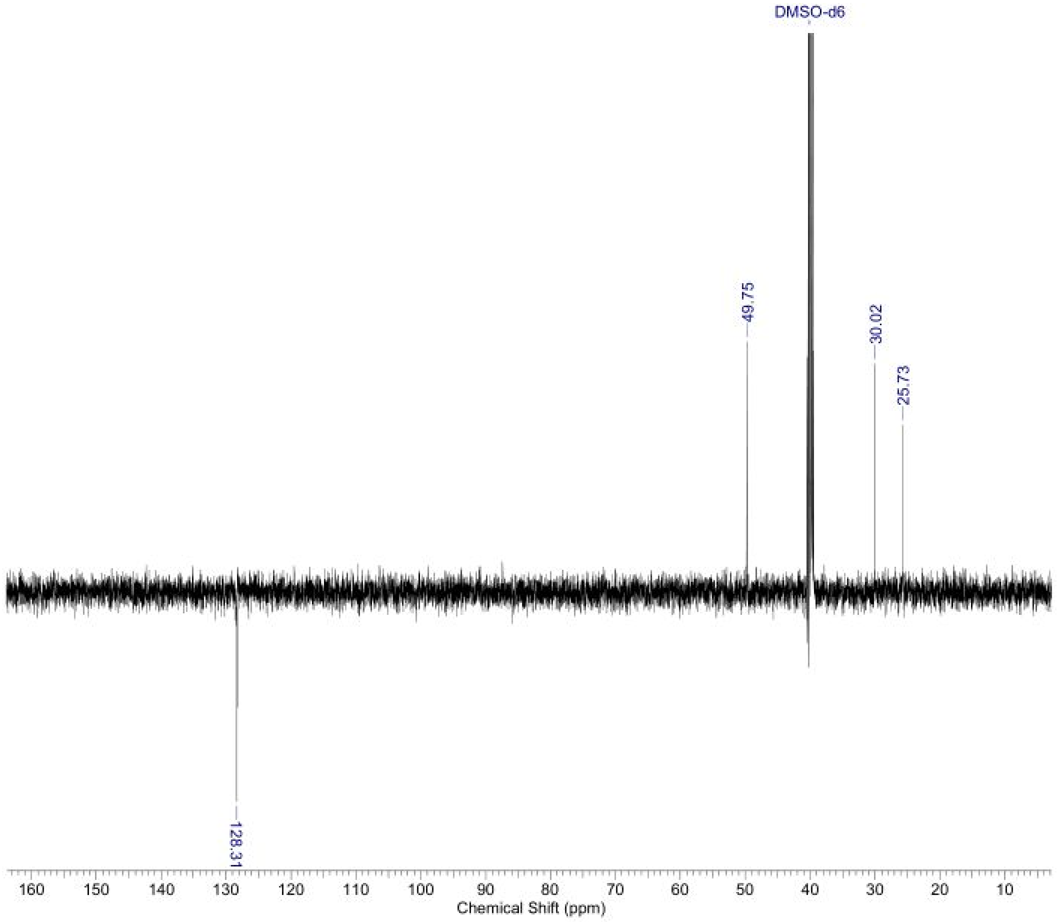

Figure S19. ^{13}C-NMR DEPT-135 of **8** in DMSO-d_6.

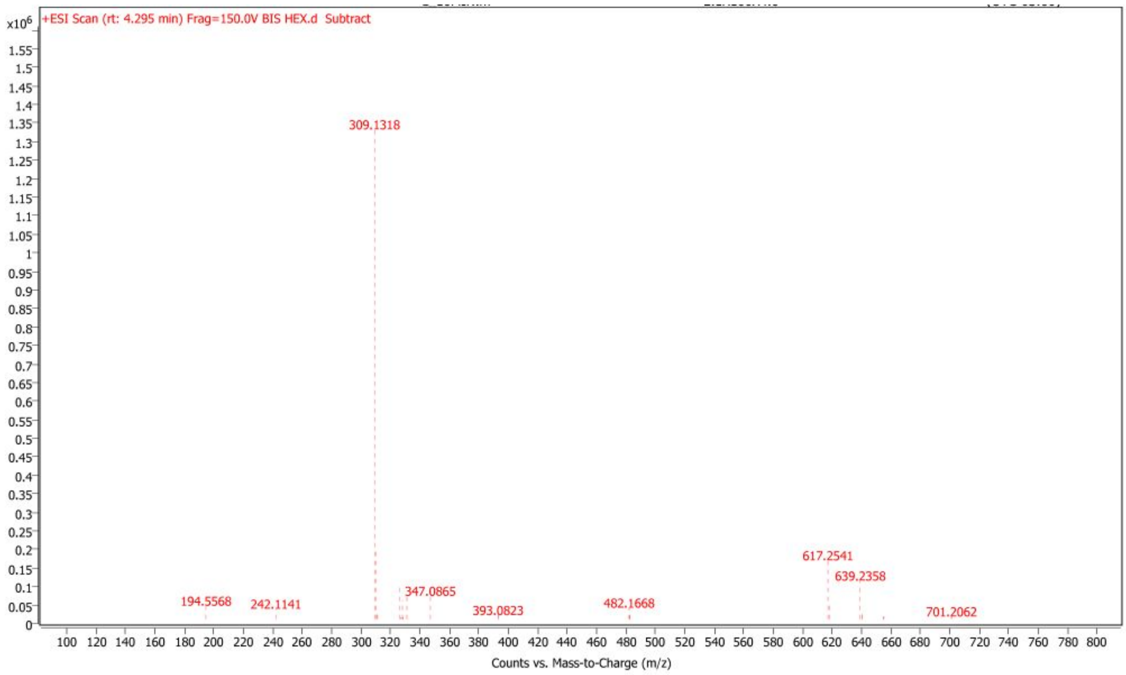

Figure S20. HRMS-TOF (MS+) of **8**.

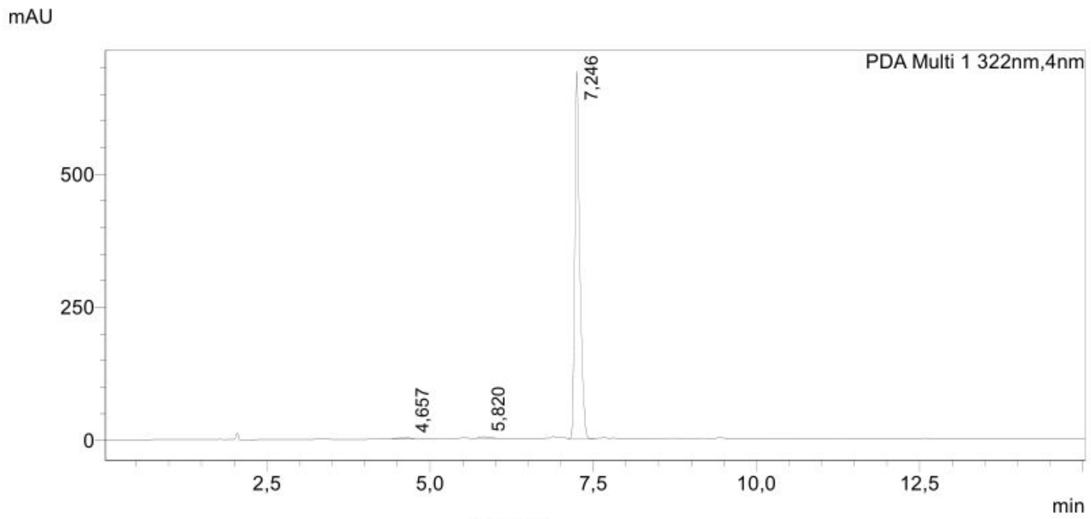

Peak Table

PDA Ch1 322nm

Peak#	Ret. Time	Area	Height	Height%	Area%
1	4,657	25217	2115	0,304	0,676
2	5,820	27863	2900	0,417	0,747
3	7,246	3674933	690377	99,279	98,576
Total		3728014	695392	100,000	100,000

Figure S21. HPLC Chromatogram of **8**.

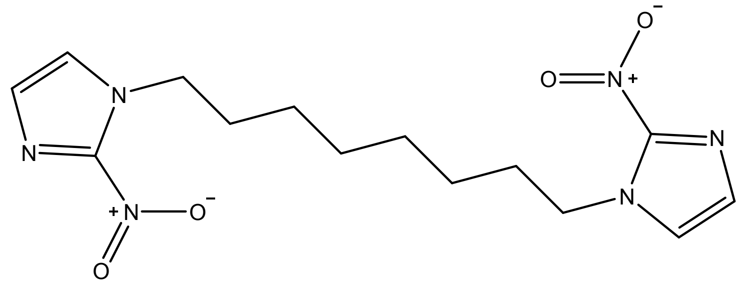

1,8-bis(2-nitro-1*H*-imidazol-1-yl)octane
Chemical Formula: $C_{14}H_{20}N_6O_4$
Exact Mass: 336,15
Molecular Weight: 336,35
m/z: 336.15 (100.0%), 337.16 (15.1%), 337.15 (2.2%), 338.16 (1.1%)
Elemental Analysis: C, 49.99; H, 5.99; N, 24.99; O, 19.03

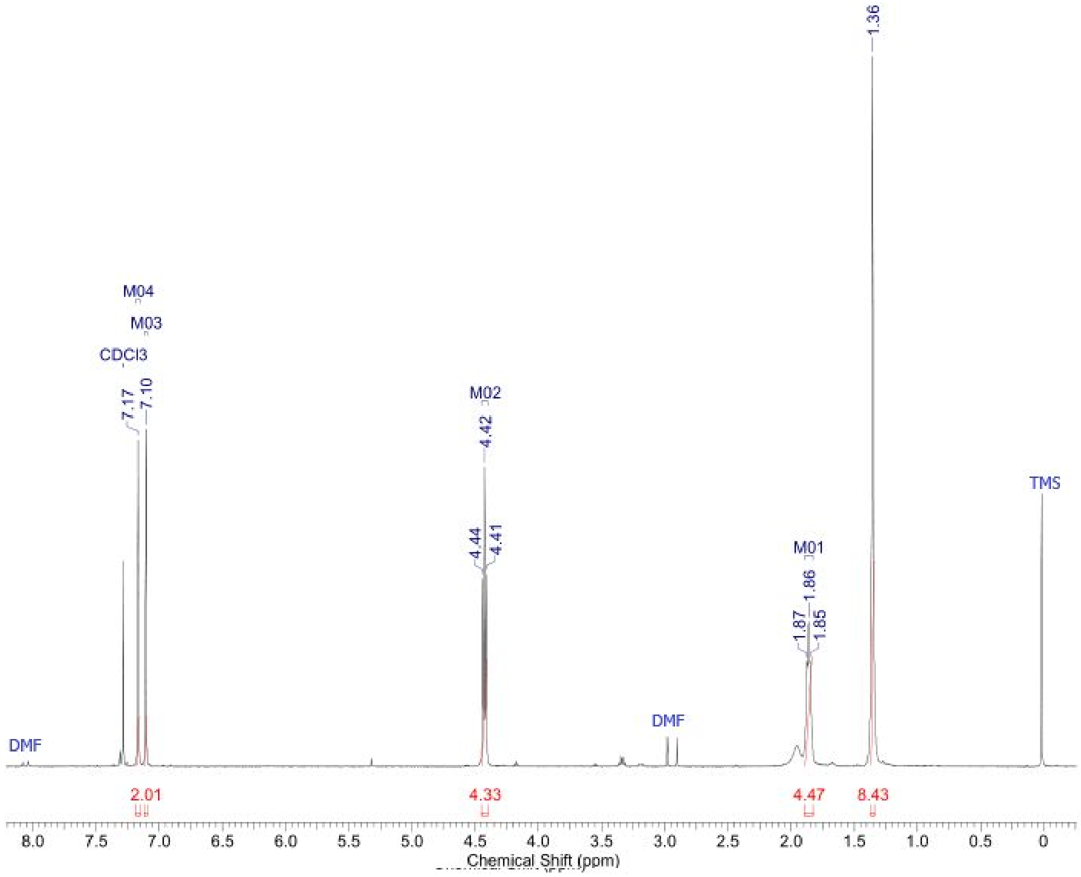

Figure S22. ^{1}H-NMR of **9** in $CDCl_3$.

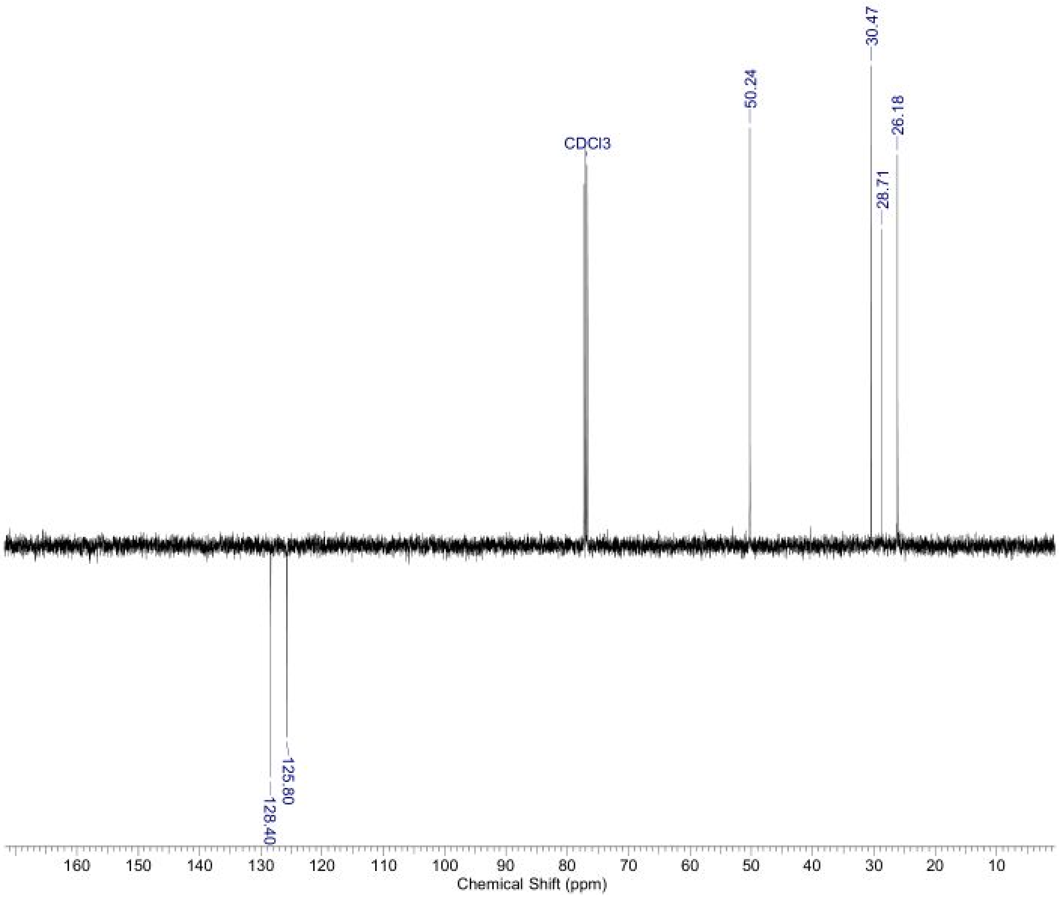

Figure S23. ^{13}C-NMR DEPT-135 of **9** in $CDCl_3$.

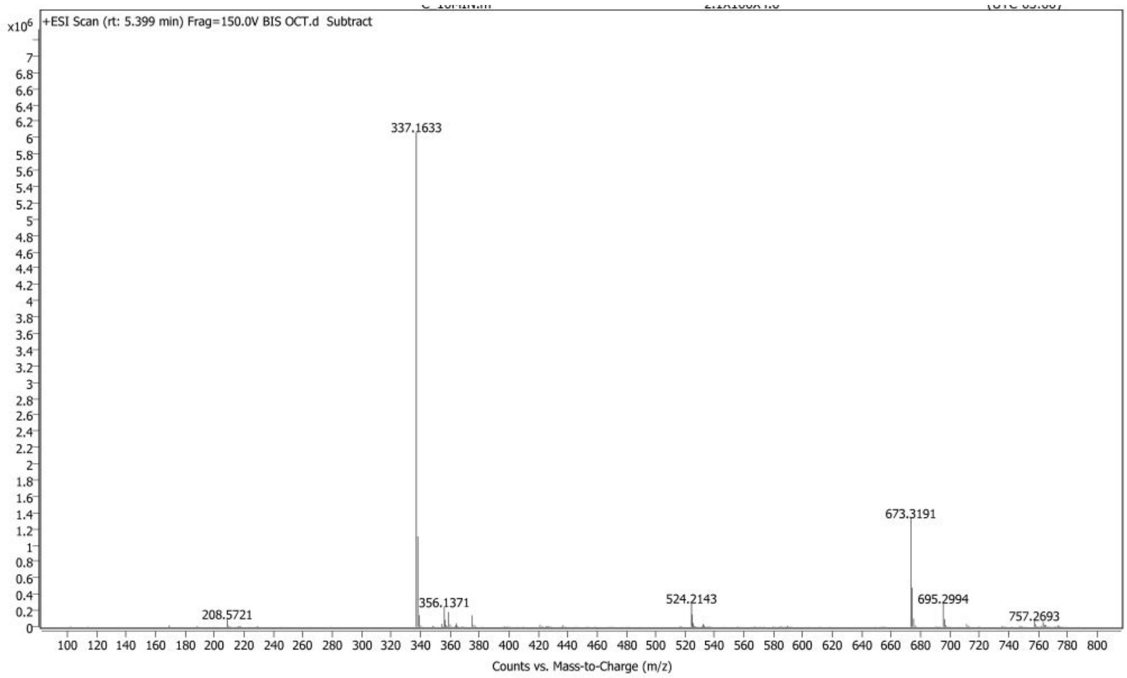

Figure S24. HRMS-TOF (MS+) of **9**.

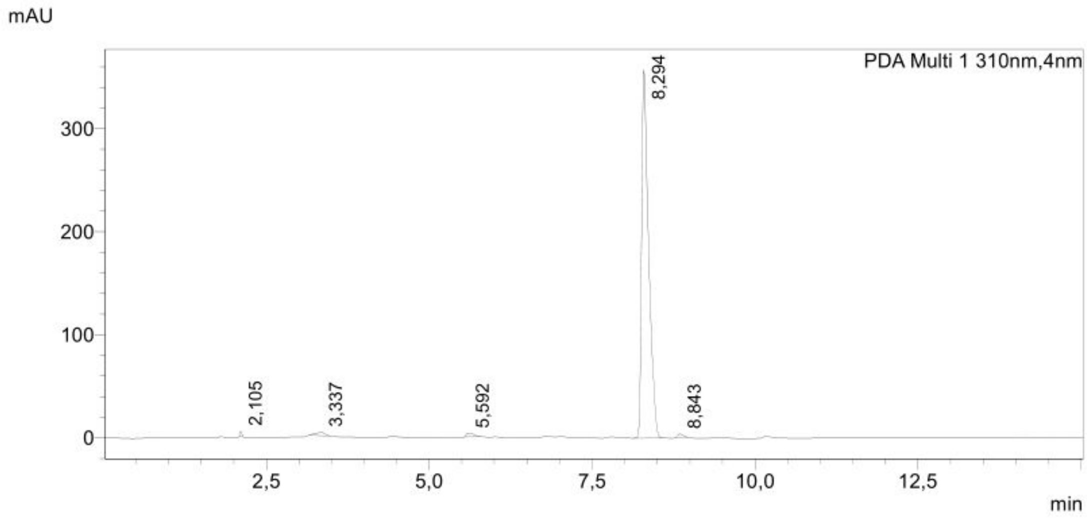

Peak Table

PDA Ch1 310nm

Peak#	Ret. Time	Area	Area%
1	2,105	8166	0,296
2	3,337	33556	1,217
3	5,592	27957	1,014
4	8,294	2667948	96,763
5	8,843	19559	0,709
Total		2757186	100,000

Figure S25. HPLC Chromatogram of **9**.

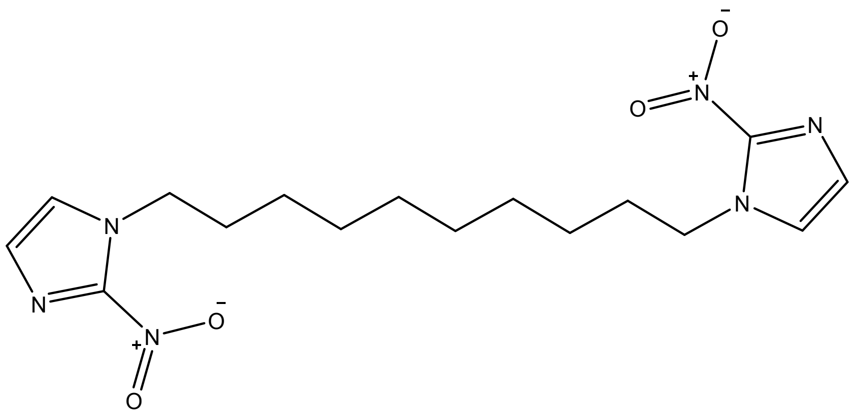

1,10-bis(2-nitro-1*H*-imidazol-1-yl)decane
Chemical Formula: $C_{16}H_{24}N_6O_4$
Exact Mass: 364,19
Molecular Weight: 364,41
m/z: 364.19 (100.0%), 365.19 (17.3%), 365.18 (2.2%), 366.19 (1.4%)
Elemental Analysis: C, 52.74; H, 6.64; N, 23.06; O, 17.56

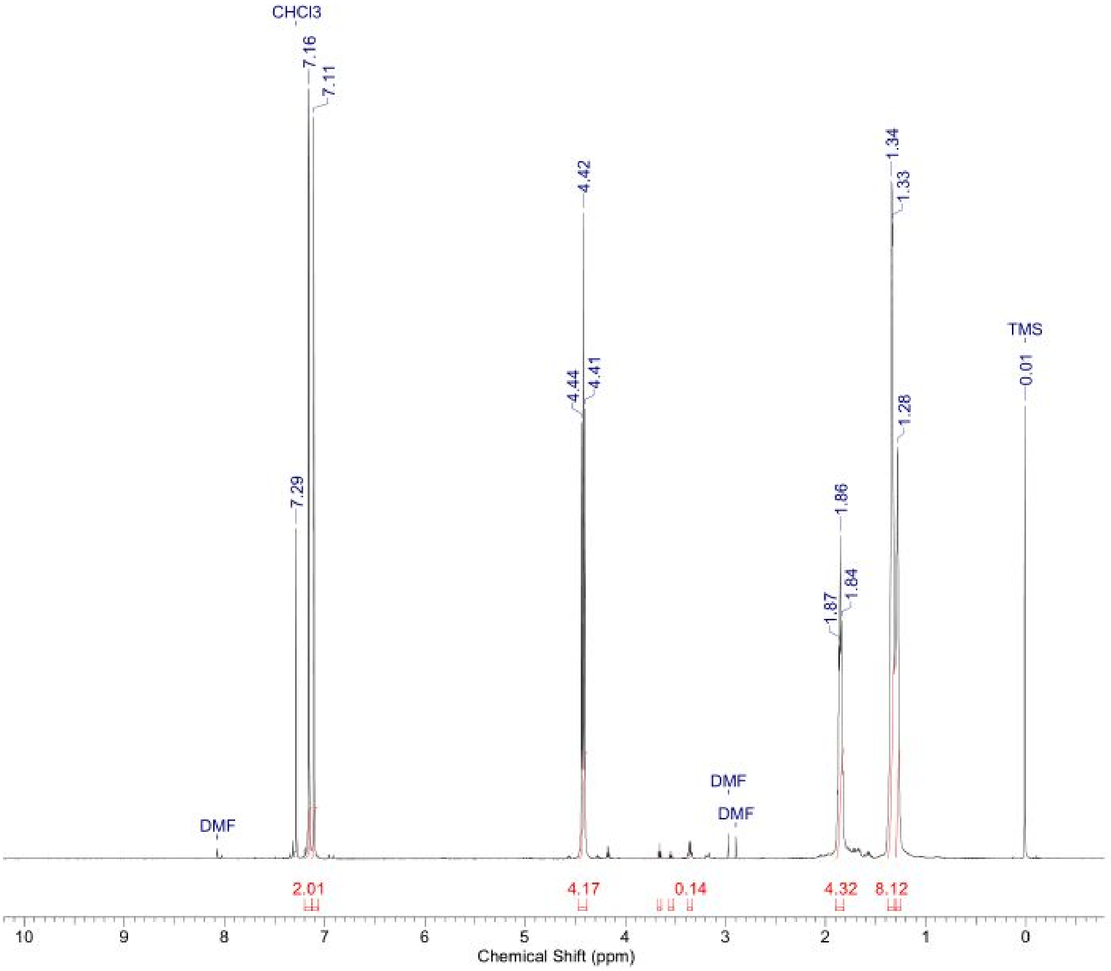

Figure S26. ^{1}H-NMR of **10** in $CDCl_3$.

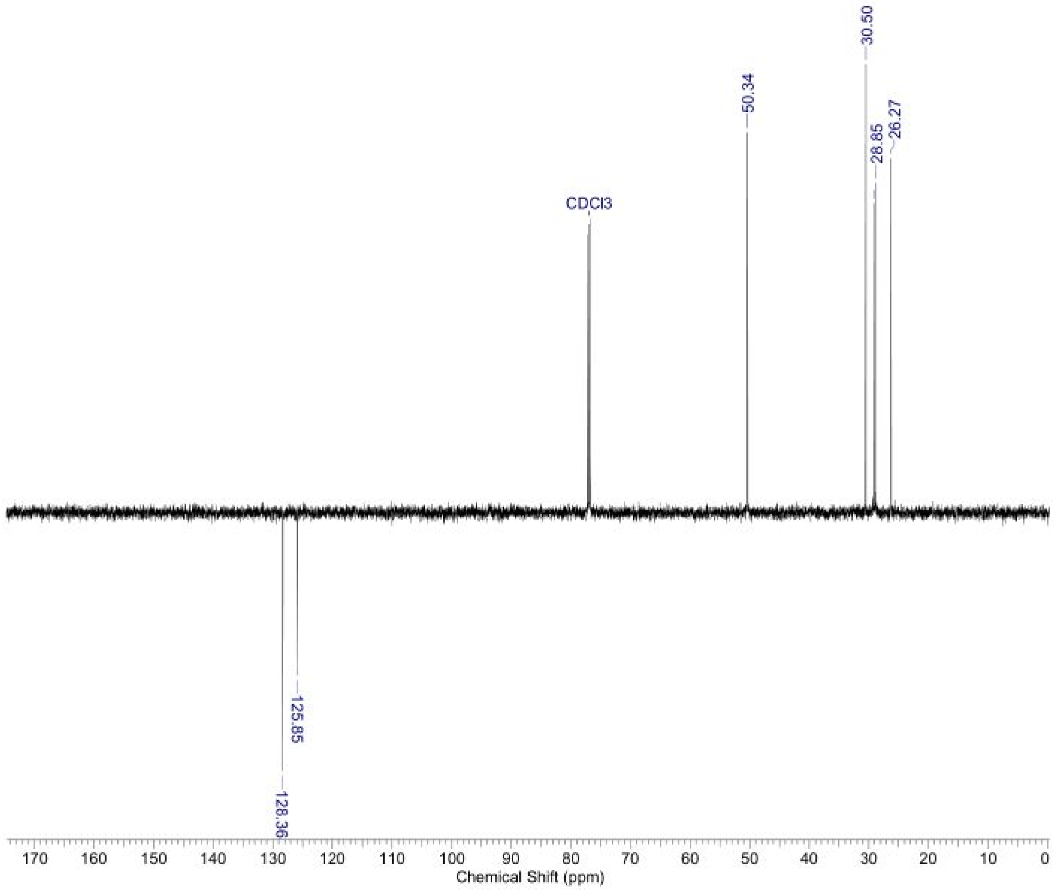

Figure S27. ^{13}C-NMR DEPT-135 of **10** in $CDCl_3$.

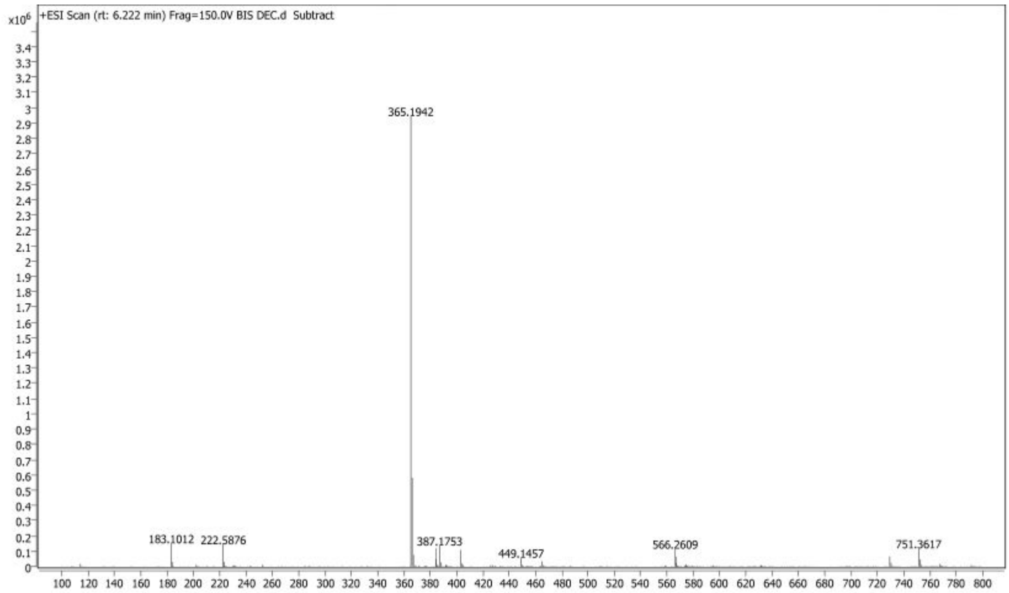

Figure S28. HRMS-TOF (MS+) of **10**.

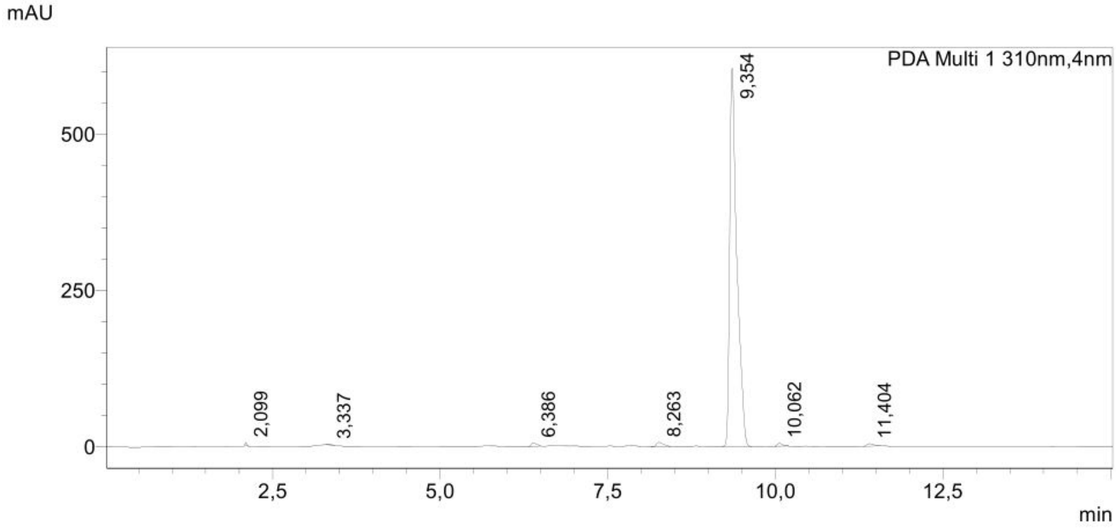

Peak Table

PDA Ch1 310nm

Peak#	Ret. Time	Area	Area%
1	2,099	5718	0,122
2	3,337	6150	0,131
3	6,386	34075	0,728
4	8,263	48285	1,032
5	9,354	4529335	96,804
6	10,062	26016	0,556
7	11,404	29270	0,626
Total		4678849	100,000

Figure S29. HPLC Chromatogram of **10**.

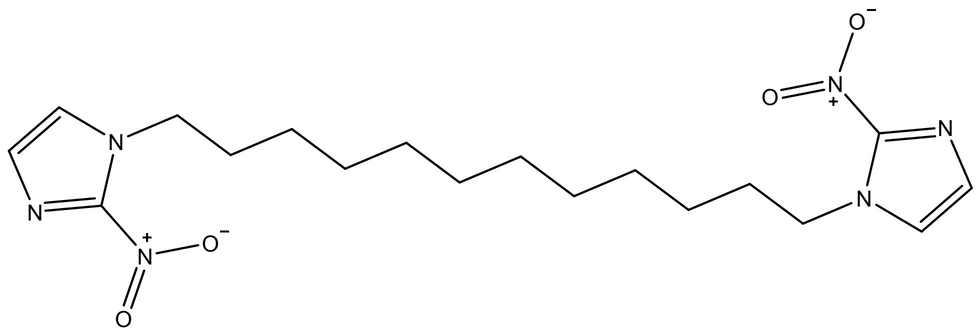

1,12-bis(2-nitro-1*H*-imidazol-1-yl)dodecane
Chemical Formula: $C_{18}H_{28}N_6O_4$
Exact Mass: 392,22
Molecular Weight: 392,46
m/z: 392.22 (100.0%), 393.22 (19.5%), 393.21 (2.2%), 394.22 (1.8%)
Elemental Analysis: C, 55.09; H, 7.19; N, 21.41; O, 16.31

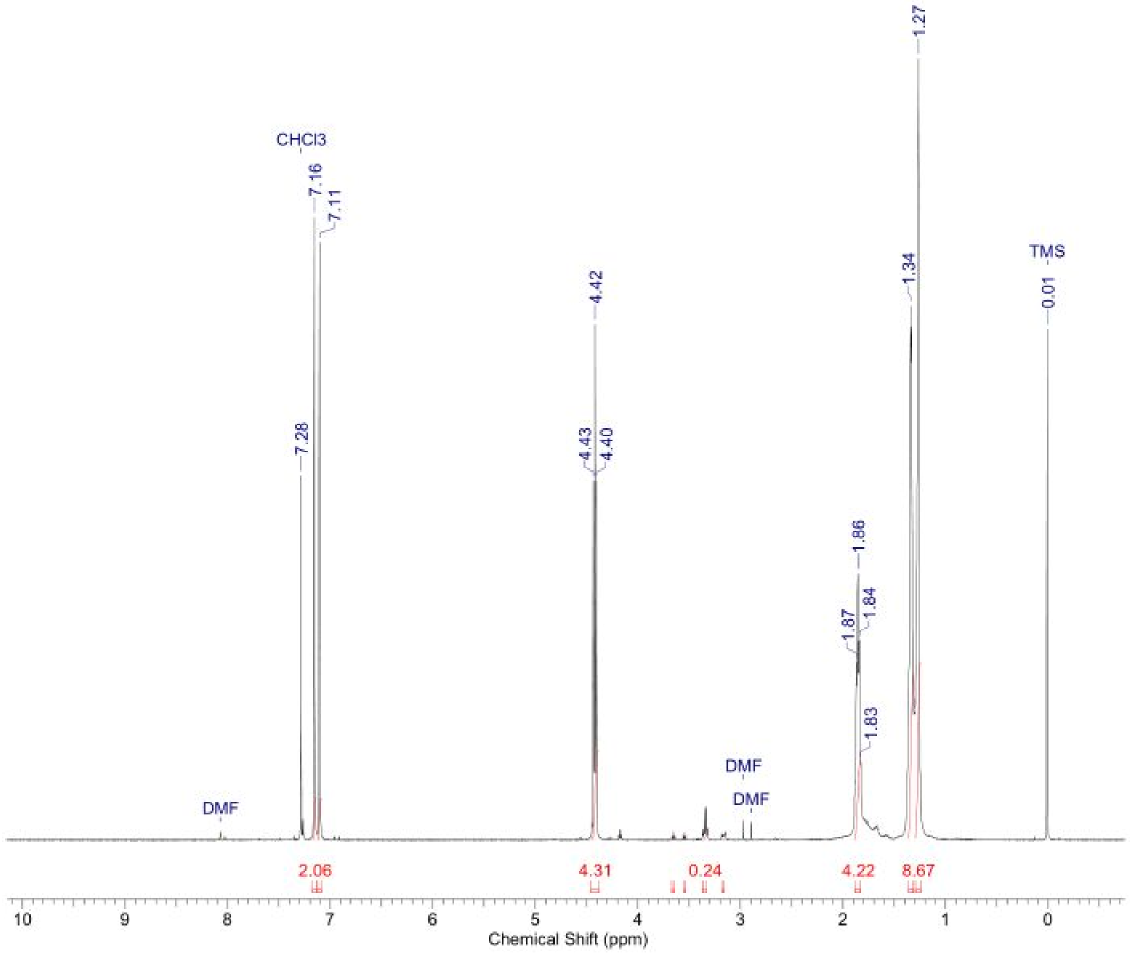

Figure S30. ^{1}H-NMR of **11** in $CDCl_3$.

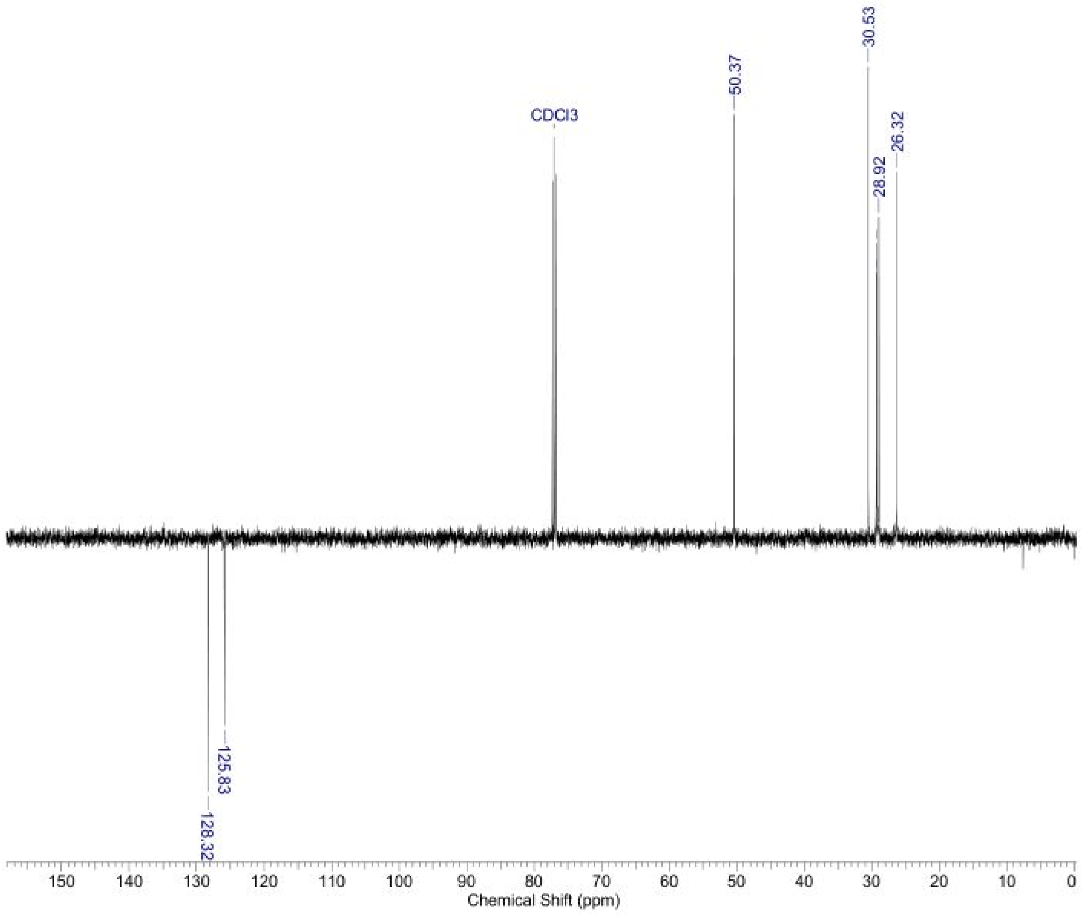

Figure S31. ^{13}C-NMR DEPT-135 DE **11** in $CDCl_3$.

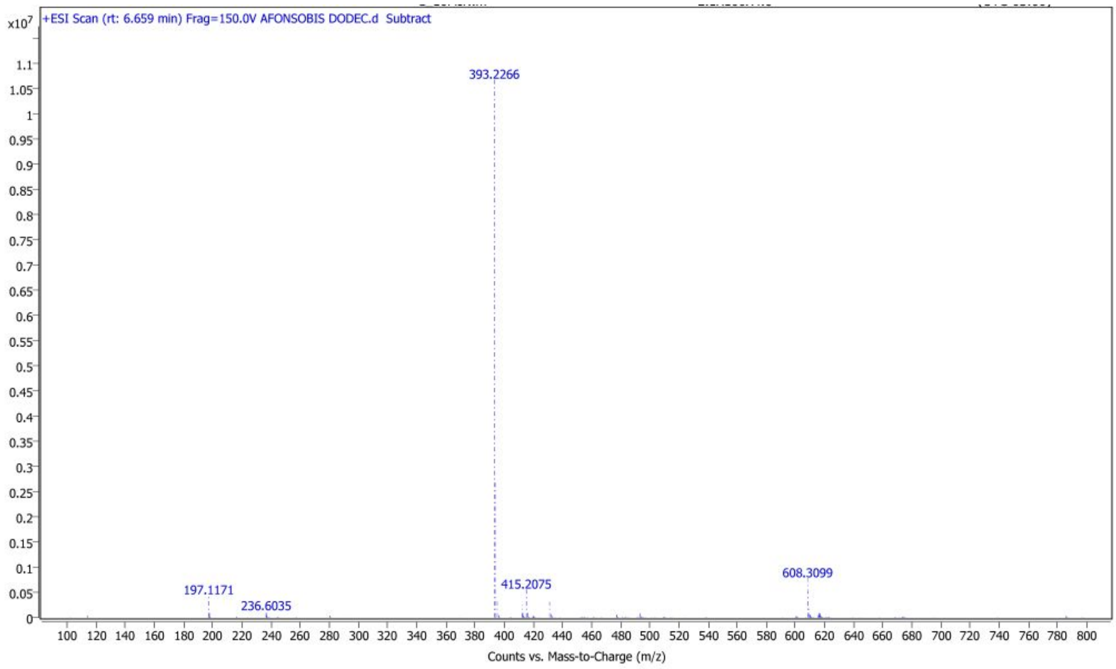

Figure S32. HRMS-TOF (MS+) of **11**.

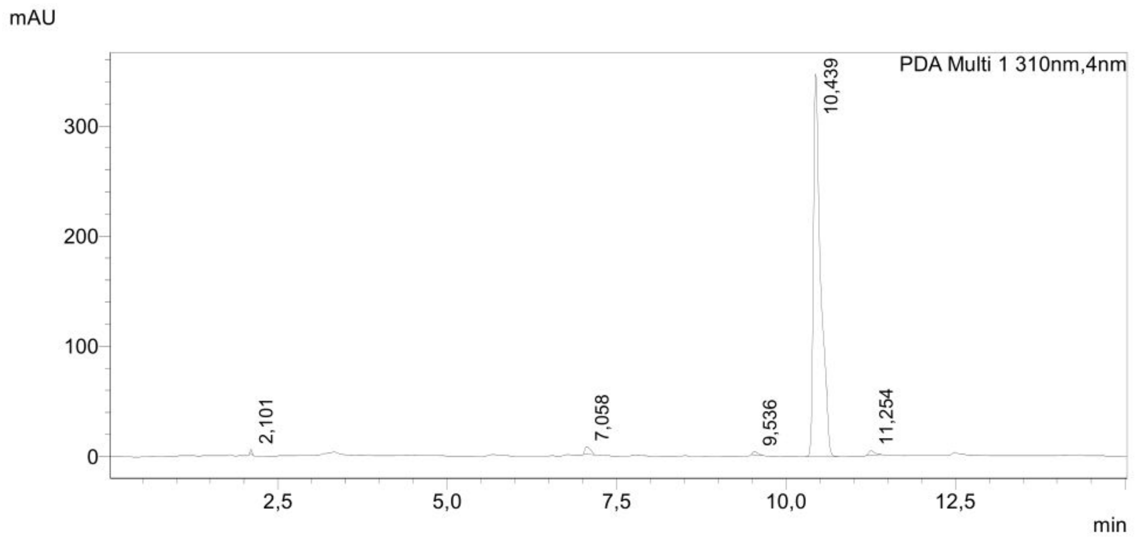

Peak Table

PDA Ch1 310nm

Peak#	Ret. Time	Area	Area%
1	2,101	6008	0,225
2	7,058	36764	1,376
3	9,536	14511	0,543
4	10,439	2592177	97,035
5	11,254	21920	0,821
Total		2671381	100,000

Figure S33. HPLC Chromatogram of **11**.

Computational details for the structural-energy minimization of compound 3

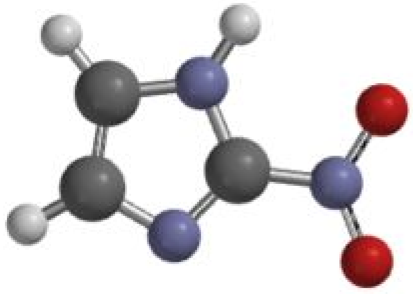

```
SPARTAN '14 MECHANICS PROGRAM: (Win/64b) Release 1.1.4


Frequency Calculation


Reason for exit: Successful completion
Mechanics CPU Time : .22
Mechanics Wall Time: .18

SPARTAN '14 Quantum Mechanics Driver: (Win/64b) Release 1.1.4

Job type: Geometry optimization.
Method: RB3LYP
Basis set: 6-31G(D)
Number of shells: 38
Number of basis functions: 126
Multiplicity: 1
Parallel Job: 8 threads

SCF model:
A restricted hybrid HF-DFT SCF calculation will be
performed using Pulay DIIS + Geometric Direct Minimization


Optimization:
Step Energy Max Grad. Max Dist.
1 -430.711175 0.010997 0.031386
2 -430.711653 0.004128 0.010268
3 -430.711730 0.001029 0.002249
4 -430.711735 0.000344 0.000764
5 -430.711735 0.000036 0.000180

Reason for exit: Successful completion
Quantum Calculation CPU Time : 13.30
Quantum Calculation Wall Time: 1:08.94


SPARTAN '14 Semi-Empirical Program: (Win/64b) Release 1.1.4
```

```
Semi-empirical Property Calculation

M0001

Guess from Archive
Energy Due to Solvation
Solvation Energy SM5.4/A -40.024
Memory Used: 440.80 Kb

Reason for exit: Successful completion
Semi-Empirical Program CPU Time : .23
Semi-Empirical Program Wall Time: .06


SPARTAN '14 Properties Program: (Win/64b) Release 1.1.4

Reason for exit: Successful completion
Properties CPU Time : .30
Properties Wall Time: .14
```

Computational details for the structural-energy minimization of compound 4

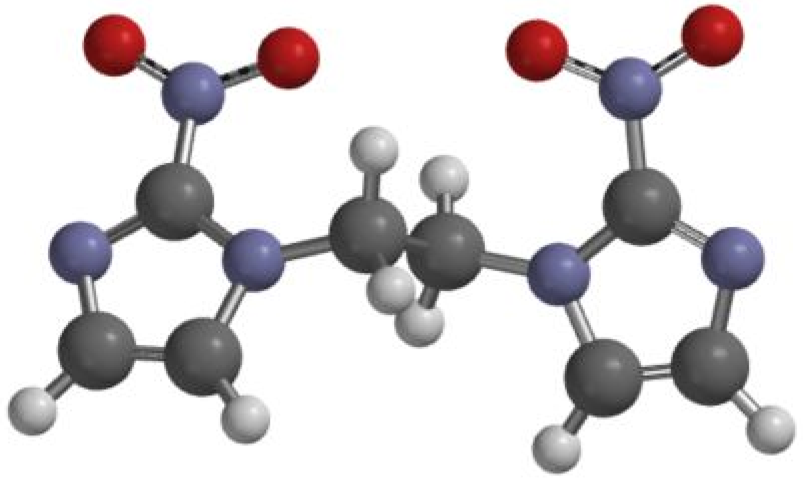

SPARTAN '14 MECHANICS PROGRAM: (Win/64b) Release 1.1.4

Frequency Calculation

Adjusted 3 (out of 78) low frequency modes

Reason for exit: Successful completion
Mechanics CPU Time : .17
Mechanics Wall Time: .22

SPARTAN '14 Quantum Mechanics Driver: (Win/64b) Release 1.1.4

Job type: Geometry optimization.
Method: RB3LYP
Basis set: 6-31G(D)
Number of shells: 88
Number of basis functions: 286
Multiplicity: 1
Parallel Job: 8 threads

SCF model:
A restricted hybrid HF-DFT SCF calculation will be
performed using Pulay DIIS + Geometric Direct Minimization

Optimization:
Step Energy Max Grad. Max Dist.
1 -938.829689 0.019855 0.144101
2 -938.833636 0.005649 0.125931
3 -938.834729 0.002939 0.120121
4 -938.835242 0.001747 0.091108
5 -938.835419 0.001299 0.028734
6 -938.835478 0.001080 0.025314
7 -938.835513 0.001047 0.034825
8 -938.835542 0.000908 0.028282
9 -938.835565 0.000501 0.039933

```
10 -938.835591 0.000688 0.035069
11 -938.835607 0.000741 0.048074
12 -938.835623 0.000514 0.107421
13 -938.835703 0.001231 0.123386 1
14 -938.836074 0.002592 0.137080 1
15 -938.836666 0.003732 0.146951 1
16 -938.837325 0.002697 0.141612
17 -938.837775 0.002215 0.102056
18 -938.837966 0.002590 0.054996
19 -938.838036 0.001399 0.017779
20 -938.838048 0.000372 0.023756
21 -938.838054 0.000451 0.006427
22 -938.838056 0.000171 0.005527
23 -938.838056 0.000098 0.003503

Reason for exit: Successful completion
Quantum Calculation CPU Time : 5:00.98
Quantum Calculation Wall Time: 15:50.02


SPARTAN '14 Semi-Empirical Program: (Win/64b) Release 1.1.4
Semi-empirical Property Calculation

M0001

Guess from Archive
Energy Due to Solvation
Solvation Energy SM5.4/A -56.689
Memory Used: 2.871 Mb

Reason for exit: Successful completion
Semi-Empirical Program CPU Time : .30
Semi-Empirical Program Wall Time: .11


SPARTAN '14 Properties Program: (Win/64b) Release 1.1.4

Reason for exit: Successful completion
Properties CPU Time : .59
Properties Wall Time: .43
```

Computational details for the structural-energy minimization of compound 5

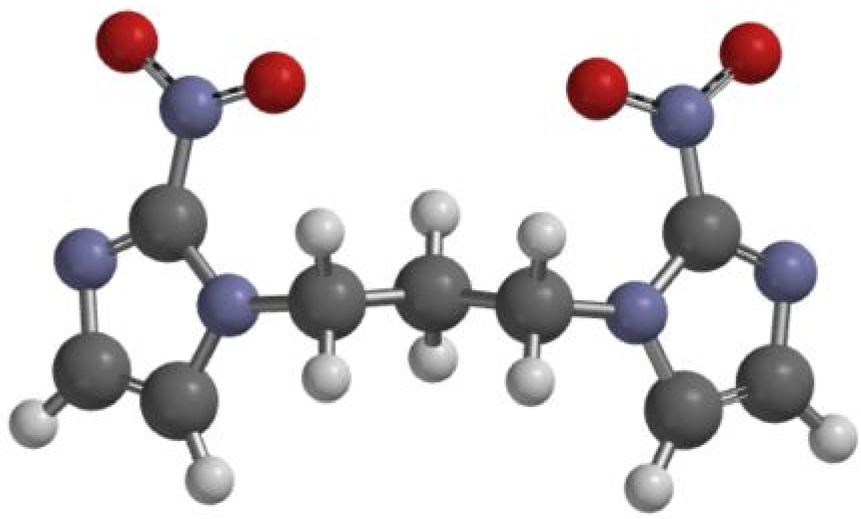

SPARTAN '14 MECHANICS PROGRAM: (Win/64b) Release 1.1.4

Frequency Calculation

Adjusted 4 (out of 87) low frequency modes

Reason for exit: Successful completion
Mechanics CPU Time : .28
Mechanics Wall Time: .05

SPARTAN '14 Quantum Mechanics Driver: (Win/64b) Release 1.1.4

Job type: Geometry optimization.
Method: RB3LYP
Basis set: 6-31G(D)
Number of shells: 96
Number of basis functions: 305
Multiplicity: 1
Parallel Job: 8 threads

SCF model:
A restricted hybrid HF-DFT SCF calculation will be
performed using Pulay DIIS + Geometric Direct Minimization

Optimization:
Step Energy Max Grad. Max Dist.
1 -978.150395 0.019163 0.127868
2 -978.153804 0.005767 0.209252
3 -978.154502 0.002903 0.175777
4 -978.154709 0.002282 0.107743
5 -978.154784 0.001211 0.020009
6 -978.154817 0.000435 0.016430
7 -978.154820 0.000231 0.008725

```
8 -978.154821 0.000126 0.008520

Reason for exit: Successful completion
Quantum Calculation CPU Time : 2:11.39
Quantum Calculation Wall Time: 6:14.04


SPARTAN '14 Semi-Empirical Program: (Win/64b) Release 1.1.4
Semi-empirical Property Calculation

M0001

Guess from Archive
Energy Due to Solvation
Solvation Energy SM5.4/A -71.084
Memory Used: 3.501 Mb

Reason for exit: Successful completion
Semi-Empirical Program CPU Time : .28
Semi-Empirical Program Wall Time: .13


SPARTAN '14 Properties Program: (Win/64b) Release 1.1.4

Reason for exit: Successful completion
Properties CPU Time : 1.06
Properties Wall Time: .66
```

Computational details for the structural-energy minimization of compound 6

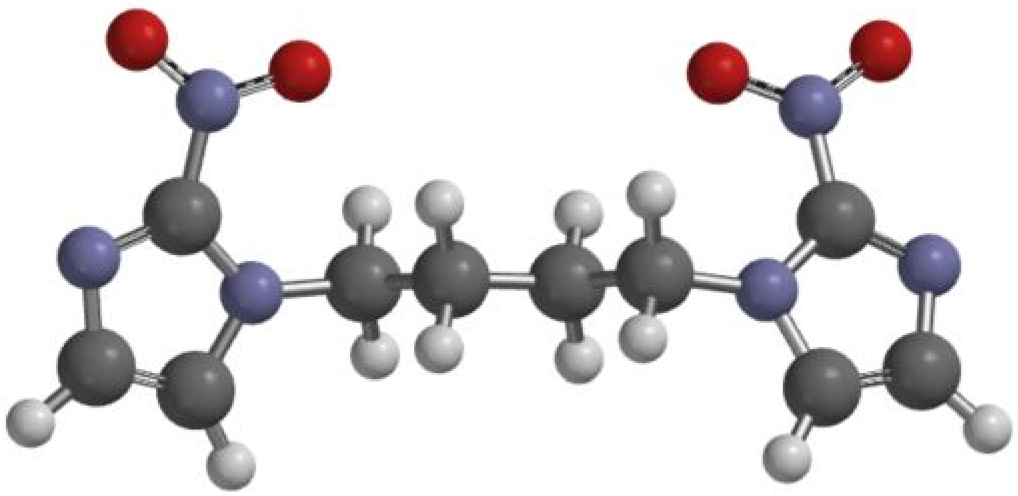

SPARTAN '14 MECHANICS PROGRAM: (Win/64b) Release 1.1.4

Frequency Calculation

Adjusted 5 (out of 96) low frequency modes

Reason for exit: Successful completion
Mechanics CPU Time : .20
Mechanics Wall Time: .04

SPARTAN '14 Quantum Mechanics Driver: (Win/64b) Release 1.1.4

Job type: Geometry optimization.
Method: RB3LYP
Basis set: 6-31G(D)
Number of shells: 104
Number of basis functions: 324
Multiplicity: 1
Parallel Job: 8 threads

SCF model:
A restricted hybrid HF-DFT SCF calculation will be
performed using Pulay DIIS + Geometric Direct Minimization

Optimization:
Step Energy Max Grad. Max Dist.
1 -1017.466833 0.016734 0.133198
2 -1017.470714 0.005014 0.185002
3 -1017.471471 0.003469 0.159541
4 -1017.471753 0.001605 0.102392
5 -1017.471839 0.001231 0.036964
6 -1017.471906 0.001114 0.043691
7 -1017.471943 0.000885 0.054017
8 -1017.471964 0.001056 0.034677

```
9 -1017.471972 0.001052 0.014817
10 -1017.471984 0.000691 0.036849
11 -1017.472005 0.000516 0.130286
12 -1017.472084 0.001229 0.041263
13 -1017.472097 0.001370 0.048375
14 -1017.472093 0.000606 0.019623
15 -1017.472103 0.000081 0.003966
16 -1017.472103 0.000050 0.000443

Reason for exit: Successful completion
Quantum Calculation CPU Time : 5:54.25
Quantum Calculation Wall Time: 16:24.75


SPARTAN '14 Semi-Empirical Program: (Win/64b) Release 1.1.4
Semi-empirical Property Calculation

M0001

Guess from Archive
Energy Due to Solvation
Solvation Energy SM5.4/A -62.858
Memory Used: 4.213 Mb

Reason for exit: Successful completion
Semi-Empirical Program CPU Time : .30
Semi-Empirical Program Wall Time: .10


SPARTAN '14 Properties Program: (Win/64b) Release 1.1.4

Reason for exit: Successful completion
Properties CPU Time : .73
Properties Wall Time: .49
```

Computational details for the structural-energy minimization of compound 7

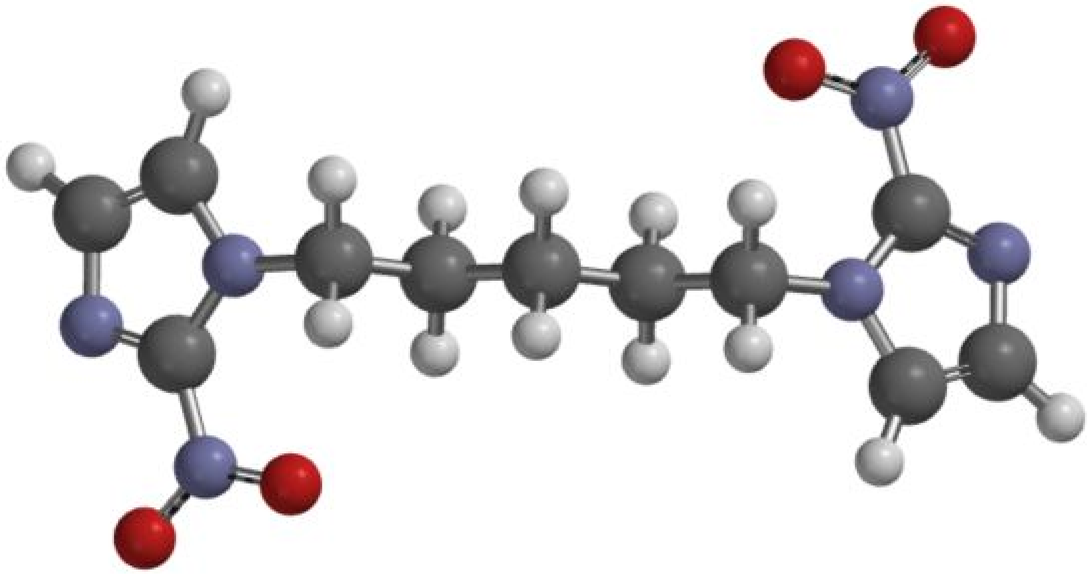

SPARTAN '14 MECHANICS PROGRAM: (Win/64b) Release 1.1.4

Frequency Calculation

Adjusted 5 (out of 105) low frequency modes

Reason for exit: Successful completion
Mechanics CPU Time : .23
Mechanics Wall Time: .23

SPARTAN '14 Quantum Mechanics Driver: (Win/64b) Release 1.1.4

Job type: Geometry optimization.
Method: RB3LYP
Basis set: 6-31G(D)
Number of shells: 112
Number of basis functions: 343
Multiplicity: 1
Parallel Job: 8 threads

SCF model:
A restricted hybrid HF-DFT SCF calculation will be performed using Pulay DIIS + Geometric Direct Minimization

Optimization:

Step	Energy	Max Grad.	Max Dist.
1	-1056.783204	0.022191	0.111752
2	-1056.787111	0.005661	0.214324
3	-1056.787861	0.004179	0.122092
4	-1056.788136	0.001980	0.100072
5	-1056.788295	0.001724	0.067581
6	-1056.788407	0.001013	0.057448

```
7 -1056.788462 0.001288 0.029248
8 -1056.788474 0.000362 0.017094
9 -1056.788478 0.000181 0.005493
10 -1056.788479 0.000138 0.002266
11 -1056.788479 0.000038 0.000688

Reason for exit: Successful completion
Quantum Calculation CPU Time : 4:32.30
Quantum Calculation Wall Time: 12:25.34


SPARTAN '14 Semi-Empirical Program: (Win/64b) Release 1.1.4
Semi-empirical Property Calculation

M0001

Guess from Archive
Energy Due to Solvation
Solvation Energy SM5.4/A -54.565
Memory Used: 5.012 Mb

Reason for exit: Successful completion
Semi-Empirical Program CPU Time : .28
Semi-Empirical Program Wall Time: .25


SPARTAN '14 Properties Program: (Win/64b) Release 1.1.4

Reason for exit: Successful completion
Properties CPU Time : .77
Properties Wall Time: .59
```

Computational details for the structural-energy minimization of compound 8

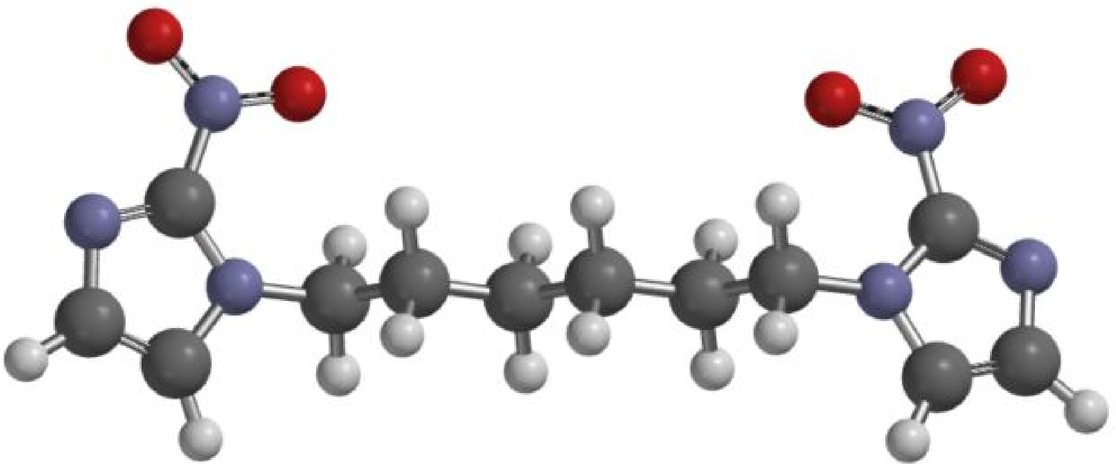

SPARTAN '14 MECHANICS PROGRAM: (Win/64b) Release 1.1.4

Frequency Calculation

Adjusted 6 (out of 114) low frequency modes

Reason for exit: Successful completion
Mechanics CPU Time : .20
Mechanics Wall Time: .11

SPARTAN '14 Quantum Mechanics Driver: (Win/64b) Release 1.1.4

Job type: Geometry optimization.
Method: RB3LYP
Basis set: 6-31G(D)
Number of shells: 120
Number of basis functions: 362
Multiplicity: 1
Parallel Job: 8 threads

SCF model:
A restricted hybrid HF-DFT SCF calculation will be performed using Pulay DIIS + Geometric Direct Minimization

Optimization:
Step Energy Max Grad. Max Dist.
1 -1096.095853 0.020044 0.111921
2 -1096.100024 0.004837 0.191151
3 -1096.100807 0.002907 0.168942
4 -1096.101112 0.002226 0.109243
5 -1096.101206 0.001314 0.037851
6 -1096.101273 0.000966 0.019995
7 -1096.101293 0.000879 0.040876
8 -1096.101316 0.000609 0.079767
9 -1096.101344 0.000890 0.025820
10 -1096.101357 0.000257 0.011966

```
11 -1096.101361 0.000252 0.005396
12 -1096.101362 0.000042 0.000685

Reason for exit: Successful completion
Quantum Calculation CPU Time : 3:18.73
Quantum Calculation Wall Time: 10:45.13


SPARTAN '14 Semi-Empirical Program: (Win/64b) Release 1.1.4
Semi-empirical Property Calculation

M0001

Guess from Archive
Energy Due to Solvation
Solvation Energy SM5.4/A -59.159
Memory Used: 5.904 Mb

Reason for exit: Successful completion
Semi-Empirical Program CPU Time : .34
Semi-Empirical Program Wall Time: .11


SPARTAN '14 Properties Program: (Win/64b) Release 1.1.4

Reason for exit: Successful completion
Properties CPU Time : .84
Properties Wall Time: .66
```

Computational details for the structural-energy minimization of compound 9

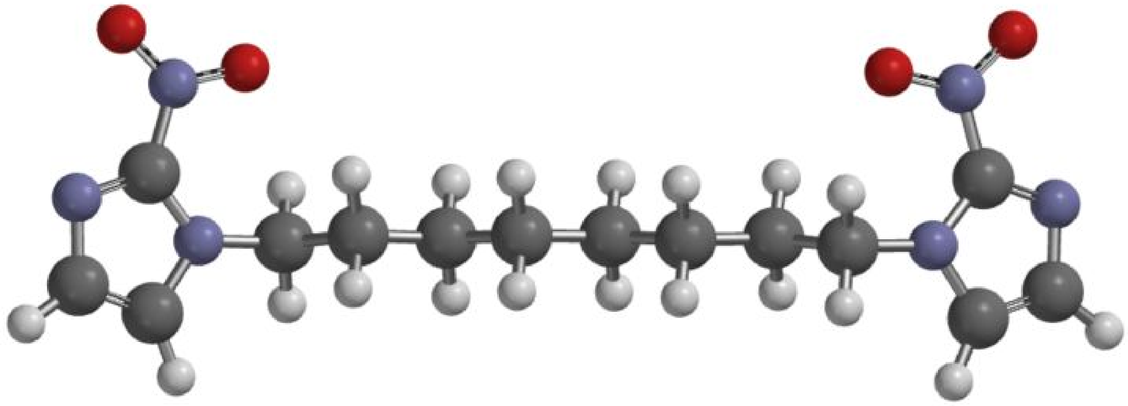

SPARTAN '14 MECHANICS PROGRAM: (Win/64b) Release 1.1.4

Frequency Calculation

Adjusted 6 (out of 132) low frequency modes

Reason for exit: Successful completion
Mechanics CPU Time : .17
Mechanics Wall Time: .05

SPARTAN '14 Quantum Mechanics Driver: (Win/64b) Release 1.1.4

Job type: Geometry optimization.
Method: RB3LYP
Basis set: 6-31G(D)
Number of shells: 136
Number of basis functions: 400
Multiplicity: 1
Parallel Job: 8 threads

SCF model:
A restricted hybrid HF-DFT SCF calculation will be
performed using Pulay DIIS + Geometric Direct Minimization

Optimization:
Step Energy Max Grad. Max Dist.
1 -1174.723386 0.021198 0.097755
2 -1174.728057 0.004547 0.180716
3 -1174.728894 0.002856 0.142910
4 -1174.729235 0.002379 0.138770
5 -1174.729358 0.001300 0.047450
6 -1174.729439 0.000741 0.018782
7 -1174.729451 0.001086 0.008804
8 -1174.729455 0.000820 0.006168
9 -1174.729460 0.000495 0.011758
10 -1174.729465 0.000309 0.017107
11 -1174.729471 0.000309 0.012213
12 -1174.729474 0.000105 0.002999

```
13 -1174.729475 0.000049 0.000844

Reason for exit: Successful completion
Quantum Calculation CPU Time : 5:29.30
Quantum Calculation Wall Time: 1:04:20.92


SPARTAN '14 Semi-Empirical Program: (Win/64b) Release 1.1.4
Semi-empirical Property Calculation

M0001

Guess from Archive
Energy Due to Solvation
Solvation Energy SM5.4/A -55.489
Memory Used: 7.984 Mb

Reason for exit: Successful completion
Semi-Empirical Program CPU Time : .52
Semi-Empirical Program Wall Time: .26


SPARTAN '14 Properties Program: (Win/64b) Release 1.1.4

Reason for exit: Successful completion
Properties CPU Time : 1.55
Properties Wall Time: 1.36
```

Computational details for the structural-energy minimization of compound 10

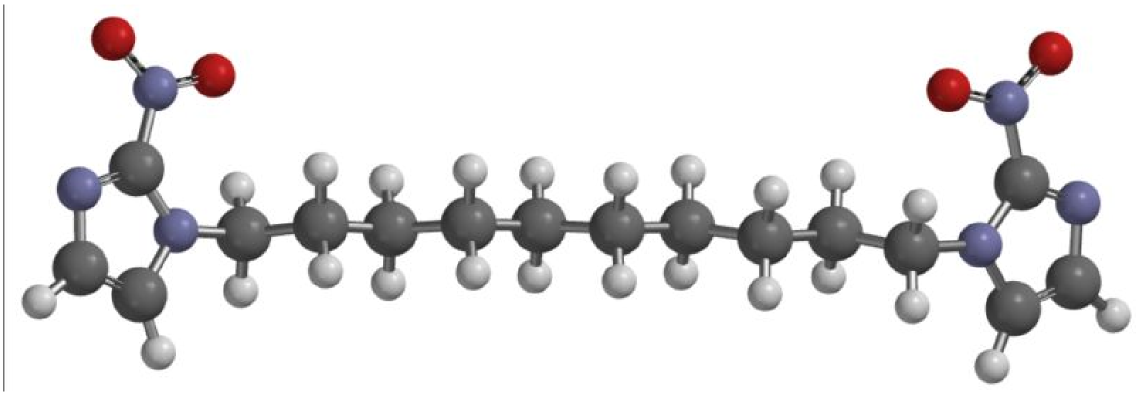

SPARTAN '14 MECHANICS PROGRAM: (Win/64b) Release 1.1.4

Frequency Calculation

Adjusted 9 (out of 150) low frequency modes

Reason for exit: Successful completion
Mechanics CPU Time : .58
Mechanics Wall Time: .14

SPARTAN '14 Quantum Mechanics Driver: (Win/64b) Release 1.1.4

Job type: Geometry optimization.
Method: RB3LYP
Basis set: 6-31G(D)
Number of shells: 152
Number of basis functions: 438
Multiplicity: 1
Parallel Job: 8 threads

SCF model:
A restricted hybrid HF-DFT SCF calculation will be
performed using Pulay DIIS + Geometric Direct Minimization

Optimization:
Step Energy Max Grad. Max Dist.
1 -1253.350689 0.027475 0.099212
2 -1253.355817 0.005722 0.143505
3 -1253.356685 0.003922 0.114736
4 -1253.356981 0.002229 0.095731
5 -1253.357087 0.001343 0.049245
6 -1253.357176 0.000863 0.037518
7 -1253.357204 0.001390 0.025107
8 -1253.357217 0.001294 0.028092
9 -1253.357239 0.001051 0.047753
10 -1253.357279 0.001236 0.038372
11 -1253.357322 0.001053 0.027056
12 -1253.357351 0.000382 0.009818

```
13 -1253.357354 0.000143 0.005974
14 -1253.357355 0.000172 0.002083

Reason for exit: Successful completion
Quantum Calculation CPU Time : 8:03.27
Quantum Calculation Wall Time: 23:53.84


SPARTAN '14 Semi-Empirical Program: (Win/64b) Release 1.1.4
Semi-empirical Property Calculation

M0001

Guess from Archive
Energy Due to Solvation
Solvation Energy SM5.4/A -52.852
Memory Used: 10.494 Mb

Reason for exit: Successful completion
Semi-Empirical Program CPU Time : .58
Semi-Empirical Program Wall Time: .33


SPARTAN '14 Properties Program: (Win/64b) Release 1.1.4

Reason for exit: Successful completion
Properties CPU Time : 1.84
Properties Wall Time: 1.59
```

Computational details for the structural-energy minimization of compound 11

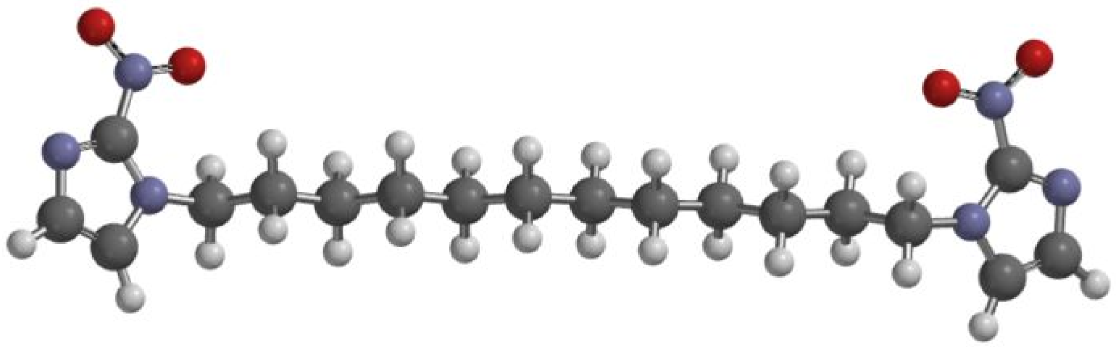

SPARTAN '14 MECHANICS PROGRAM: (Win/64b) Release 1.1.4

Frequency Calculation

Adjusted 9 (out of 168) low frequency modes

Reason for exit: Successful completion
Mechanics CPU Time : .33
Mechanics Wall Time: .10

SPARTAN '14 Quantum Mechanics Driver: (Win/64b) Release 1.1.4

Job type: Geometry optimization.
Method: RB3LYP
Basis set: 6-31G(D)
Number of shells: 168
Number of basis functions: 476
Multiplicity: 1
Parallel Job: 8 threads

SCF model:
A restricted hybrid HF-DFT SCF calculation will be
performed using Pulay DIIS + Geometric Direct Minimization

Optimization:
Step Energy Max Grad. Max Dist.
1 -1331.977626 0.032288 0.114390
2 -1331.983351 0.005984 0.184164
3 -1331.984236 0.004070 0.140416
4 -1331.984556 0.001817 0.133523
5 -1331.984674 0.001206 0.058906
6 -1331.984792 0.001517 0.066321
7 -1331.984859 0.002031 0.037227
8 -1331.984902 0.001875 0.053380
9 -1331.984973 0.001297 0.044037
10 -1331.985038 0.001243 0.040541
11 -1331.985079 0.000659 0.027550
12 -1331.985092 0.000183 0.010953
13 -1331.985094 0.000197 0.002938
14 -1331.985095 0.000071 0.002005

```
15 -1331.985095 0.000029 0.000284

Reason for exit: Successful completion
Quantum Calculation CPU Time : 9:28.16
Quantum Calculation Wall Time: 44:32.65


SPARTAN '14 Semi-Empirical Program: (Win/64b) Release 1.1.4
Semi-empirical Property Calculation

M0001

Guess from Archive
Energy Due to Solvation
Solvation Energy SM5.4/A -50.813
Memory Used: 13.471 Mb

Reason for exit: Successful completion
Semi-Empirical Program CPU Time : .66
Semi-Empirical Program Wall Time: .34


SPARTAN '14 Properties Program: (Win/64b) Release 1.1.4

Reason for exit: Successful completion
Properties CPU Time : 2.22
Properties Wall Time: 2.83
```
